# VariantMedium: sensitive and generalizable somatic point mutation calling with 3D DenseNets trained and evaluated on experimental data

**DOI:** 10.1186/s13073-026-01675-1

**Published:** 2026-06-19

**Authors:** Özlem Muslu, Thomas Bukur, Pablo Riesgo-Ferreiro, Sameesh Kher, Shaya Akbarinejad, Luis Kress, Stefania Gangi Maurici, Muhammad Nabeel Asim, Alina Henrich, Sheraz Ahmed, Andreas Dengel, Martin Löwer, Jonas Ibn-Salem, Ugur Sahin

**Affiliations:** 1 TRON – Translational Oncology at the University Medical Center of Johannes Gutenberg, University gGmbH, TRON gGmbH, Freiligrathstraße 12, Mainz, D-55131 Germany; 2 Faculty of Biology, Johannes Gutenberg University of Mainz, Mainz, Germany; 3 Deutsches Forschungszentrum für künstliche Intelligenz GmbH, Kaiserslautern, Germany; 4 Rheinland-Pfälzische Technische Universität Kaiserslautern-Landau, Kaiserslautern, Germany; 5 Johannes Gutenberg University Mainz, Mainz, Germany; 6 BioNTech Corporation, BioNTech SE, An der Goldgrube 12, Mainz, 55131 Germany

**Keywords:** Somatic variant calling, Single nucleotide variant, Somatic SNV, Somatic mutation, Tumor-normal sequencing, Somatic call benchmarking, Precision oncology, Deep learning, Machine learning, Open source software

## Abstract

**Background:**

Accurately identifying somatic variants from genomic sequencing is crucial for understanding and treating cancer. Previously, methods based on statistics and heuristics, as well as methods based on machine learning were proposed for somatic single nucleotide variant (SNV) calling from matched tumor-normal data, but they suffer from low sensitivity especially in certain genomic regions.

**Methods:**

Here we present VariantMedium, a somatic variant caller that combines a tree-based classifier with a 3D densely connected convolutional network (DenseNet) architecture. We trained and evaluated our model on experimentally confirmed variant data and improved it with an active learning strategy by experimentally confirming the predicted variants via targeted deep sequencing experiments. Overall, we used 336,839 variants from 2,956 samples with whole exome or genome sequencing for training and validation, and 118,887 variants from two independent studies with deep sequencing data for evaluation and benchmarking.

**Results:**

VariantMedium shows highest sensitivity amongst benchmarked callers and achieves similar or better F1 scores in SNV calling. Its performance is particularly pronounced on genomic regions characterized by high sequencing error rates, achieving higher F1 scores than Mutect2 and Strelka2.

**Conclusions:**

Our results demonstrate the strength of combining machine learning with high-quality experimental confirmation, enabling accurate somatic mutation detection even in low-mappability regions. We provide VariantMedium (https://github.com/TRON-Bioinformatics/VariantMedium) as an end-to-end pipeline to advance somatic mutation calling for precision medicine.

**Supplementary Information:**

The online version contains supplementary material available at 10.1186/s13073-026-01675-1.

## Background

Accumulation of somatic mutations is one of the fundamental characteristics of cancer [1–3]. Accurately identifying somatic variants in next-generation sequencing data of matched tumor and normal samples is not only essential to understand cancer, cancer evolution, tumorigenesis, and prognosis, but also to select targeted treatments and to design personalized therapies [4–6]. Identifying individual variants from aligned sequencing data is performed by comparing tumor and normal genomic data, however it is challenging to differentiate true variants from errors due to multiple biological and technical factors including tumor/normal contamination, tumor subclonality, copy number variations, DNA degradation, sequencing errors, and duplicated or ambiguously mapped reads especially in the presence of repetitive sequences [7–11]. More accurate detection of variants can improve the selection of the most suitable treatment for a patient and broaden the landscape of individual targets (e.g. neoantigens) for personalized immunotherapies [12, 13].

There is a plethora of methods optimizing somatic SNV calling from matched tumor-normal short read sequencing data, primarily using statistical methods and heuristic filtering [14–22]. While these variant callers are successful and frequently used, their assumptions on statistical independence do not hold, they might rely on external data sources (e.g. common germline variants), and they perform better in certain genomic regions than others with high/low GC content or repetitive sequences. To improve the accuracy of these approaches, some researchers leverage ensemble methods where they algorithmically integrated orthogonal mutation callers to maximize the sensitivity [23–31] or use variant refinement tools to eliminate false positives [32–36]. More recently, machine learning (ML)-based callers were proposed to increase precision, sensitivity and adaptability with the premise of nonlinear modeling of variant properties [37–45]. These methods were typically evaluated using simulated data or with cell lines or high-purity tumor samples. Given the heterogeneity of somatic mutations, a more suitable performance assessment requires a patient-derived data set spanning diverse tumor types and purities with systematically designed experimental confirmation of somatic variant calls, such as PCAWG-Pilot63 dataset [24].

Here we propose VariantMedium, a deep learning–based workflow for sensitive somatic SNV calling trained on experimentally confirmed variants from cell lines and tumor samples. VariantMedium takes matched tumor–normal whole exome sequencing (WES) or whole genome sequencing (WGS) data and produces a sensitive list of mutation calls using an extremely randomized trees (ExtraTrees) classifier for candidate selection followed by a 3D DenseNet for final classification. We benchmark the method against multiple other callers on two independent datasets with targeted deep sequencing confirmation, spanning diverse tumor types, sample purities, sequencing centers, and sequencing protocols.

## Methods

### Dataset overview

We generated or obtained in total five datasets (cohorts) with sequencing data and ground truth labels of somatic variants from different sources. Three datasets were used for training and two were reserved for evaluation (Table 1). To train the model with high-quality labels, we applied targeted deep sequencing confirmation experiments for five cell lines and performed two additional rounds of targeted sequencing on three of these cell lines. To increase training data diversity and improve generalizability, we included six additional cell lines and consensus calls (Additional file 1: Table S1) and incorporated two experimentally confirmed resources: AML31 [46], which adds WGS-based labels outside exome regions, and TCGA-MC3 [47], which introduces broader variation in tumor type, purity, and clonality. Generalizability and robustness were assessed on independent test sets with data from PCAWG and SEQC2, which have different sequencing characteristics to the datasets used for training and validation (Table 1). The train, validation, and test data splits were defined at the dataset level (Additional file 1: Table S2), such that variants from a given dataset were assigned exclusively to one split. The sources of ground-truth labels for variants are described below and in Additional file 1: Table S3. 

**Table 1:** The training, validation, and test datasets with their source, use and characteristics. Source refers to the study in which the used targets were deep sequenced, samples and labels columns show the number of samples used in this publication. Median depth of coverages of normal and tumor are calculated over the variants with labels using a custom script. Read length depicts the median read length over different samples in the study

Source	Used for	Technology	Samples	Labels	Depth of coverage (normal)	Depth of coverage (tumor)	Read length
Cell lines	Training/validation	WES	11	140,903	137x	134x	51 bp
AML31	Training	WGS	1	8345	127x	325x	100 bp
TCGA	Training/validation	WES	2944	158,682	76x	70x	76 bp
PCAWG	Test	WGS	37	106,749	37x	52x	101 bp
SEQC2	Test	WES	12	12,138	60x	49x	125 bp

### Cell line data

We generated sequencing and confirmation data for 11 cell lines pairs of matched tumor and normal origin to train and fine tune the VariantMedium models by deep sequencing the calls of our preliminary models and using the targeted deep sequencing results to retrain them. Cell lines originated from public repositories or previously published patient-derived models and were obtained from ATCC, MSKCC, or the laboratory of T. Wölfel at the University Medical Center Mainz. ATCC-derived lines included breast cancer, small cell lung cancer, melanoma and neuroendocrine carcinoma cell lines; COLO-829/COLO-829-BL, HCC-1187/HCC-1187-BL, HCC-1937/HCC-1937BL, HCC-1954/HCC-1954-BL, NCI-H-2171/NCI-BL-2171, and NCI-H-1770/NCI-BL-1770. The SK-MEL-29 melanoma model and matched EBV-transformed control (SK-29-EBV-B) were obtained from MSKCC. Additional melanoma and pancreas cancer models (MZ-2-Mel-43/MZ-2-EBV-B, MZ-7-Mel-1/MZ-7-EBV-B, MZ-PC-1/MZ-PC-1-EBV-B, MZ-PC-2/MZ-PC-2-EBV-B) were derived from previously established patient-specific tumor–lymphocyte systems described in earlier studies by the laboratory of T. Wölfel at the University Medical Center Mainz [48–50]. We sequenced 11 matched tumor-normal samples with whole exome sequencing (WES) in two technical replicates each, and 5 matched cell lines using orthogonal targeted deep sequencing [51] (Additional file 1: Tables S1, S3). We applied targeted deep sequencing confirmation experiments for five cell lines and performed two additional rounds of targeted sequencing on three of these cell lines. To increase training data diversity and improve generalizability, we included six additional cell lines and consensus calls.

#### Whole exome sequencing, alignment, and BAM preprocessing

DNA exome capture libraries were prepared in replicates from 1,000 ng DNA using the Agilent SureSelect XT Target Enrichment System for Illumina Paired-End Sequencing Library Kit and SureSelect XT V6 Human All Exon. The gDNA was sheared and end-repaired. The ends of end-repaired DNA were adenylated and adapters are ligated. After library preparation, targets were captured by hybridization of biotinylated RNA library probes. Captured target sequences were isolated using streptavidin-coated magnetic beads and amplified. Sequencing libraries were further checked for quality and quantity using the Qubit dsDNA HS Assay Kit (Invitrogen) and the Agilent Bioanalyzer 2100 System with the High Sensitivity DNA kit. All libraries were sequenced in paired-end mode (2 × 50 nt) on an Illumina HiSeq 2500 platform in High Output Run Mode (TruSeq SBS V3). Two exome capture libraries were sequenced in one lane, resulting in approximately 100 million distinct sequencing read pairs per library.

We aligned the reads to the hg19 reference genome with bwa v0.5.9-r16 [52] and processed alignment files according to the GATK best practices [53], implemented in tronflow-bam-preprocessing pipeline (v1.1.0) [54], using known insertion-deletions (indels) from GATK bundle [55, 56], and dbSNP v138 [57]. BAM preprocessing includes reformatting of BAM files, marking of duplicate reads, realignment around indels, and base quality score recalibration. 

#### Variant confirmation by targeted deep sequencing

We performed targeted deep sequencing in three batches on the variants we identified using the somatic variant callers outlined in Additional file 1: Table S4. We formatted all variants of interest into a standardized representation [58] and generated qPCR primers for the variant positions as described previously [12].

Amplicon generation was done using custom designed primers (5 μm) via PCR with 10ng input DNA, 35 cycles and HotStarTaq Master Mix Kit from Qiagen. Quality control was performed using Agilent Fragment Analyzer. The DNA amplicons from one cell line sample were then equimolar pooled together to prepare NGS libraries. The libraries were prepared from 50 ng DNA by using the NEB Next Ultra II DNA Library Prep Kit for Illumina. The workflow consists of end repair, phosphorylation and A-tailing, adapter ligation, size selection, PCR enrichment of adaptor-ligated DNA (first run: 8 cycles, second and third runs: 7 cycles), and bead clean up. Final quality control was done using the Qubit 3 fluorometer and Agilent Bioanalyzer 2100. The libraries were sequenced 300 cycles in paired-end mode (2 × 150 nt) on Illumina’s MiSeq with a v3 flow cell reagent kit resulting in approximately 5 million distinct sequencing reads per library. The reads were aligned to hg19 reference genome using bwa v0.7.9a-isis-1.0.2. 

#### Assignment of ground-truth labels in cell lines

We determined the labels somatic, germline, or no mutation for each candidate variant from one of three sources. The primary source was somatic variants confirmed by targeted deep sequencing. To address uncertainty for candidates lacking targeted deep sequencing data (due to failures in library preparation/sequencing, absence from call sets, location outside target exome regions, or, most commonly, the unavailability of targeted sequencing for the respective cell line), we extended the label set using a consensus approach. As a third source of labeling, we included DeepVariant calls to distinguish germline variants from reference positions with no evidence of variation. Since this dataset is used for training, we excluded candidate variants when they were called by a variant caller but could not be reliably classified by any of the three label sources. 

#### Targeted deep sequencing labels

After sequencing and alignment, we identified the variants in the targeted regions with CallSomaticVariants v3.5.4.0 command in resequencing workflow v2.0.0 in MiSeq equipment (-f 0 -fo True -b 20 -q 100 -c 0 -s 0.5 -a 20 -F 20 -gVCF True). We only kept the variants with PASS filter. We removed the variants that did not have sufficient coverage (1000x) in normal or tumor from the dataset. In the remaining set of variants, we labeled them as follows: “no mutation” if the variant was absent in both tumor and normal samples; “somatic” if the variant was present only in the tumor; and “germline” if it was detected in both tumor and normal samples. We excluded the variants labelled as somatic but had normal evidence ((variant allele frequency (VAF) > 0.05 and coverage > 40) or (number of variant supporting reads in normal > 2) or (VAF > 0.05 and coverage > 20). 

#### Consensus labels

We labeled the candidate variants from the cell lines HCC-1187, MZ-2-Mel-43, MZ-7-Mel-1, NCI-H-1770, NCI-H-2171, SK-MEL-29; the candidate synonymous variants from the cell lines HCC-1937, HCC-1954; and the variants that failed targeted sequencing from the cell lines COLO-829, HCC-1937, HCC-1954, MZ-PC-1, MZ-PC-2 using the following method. We first tested various combinations of Mutect2 (GATK v4.1.3) [20], Strelka2 (v2.9.9) [21], and VarScan2 (v2.4.2) [18] using the first batch of targeted deep sequencing data on cell lines COLO-829, HCC-1937, HCC-1954, MZ-PC-1, MZ-PC-2 as the truth set. We identified the following combination as the one with the best precision (>99%) over the deep sequenced variants and we chose to annotate all variants within this set as somatic consensus variants:

$$\:\left(M\cap\:S\right)\cup\:\left(M\cap\:V\right)$$

Where M denotes Mutect2 calls, S denotes Strelka2 calls, and V denotes Varscan calls, and L denotes labels. If the variants were detected by either of these callers $\:\left(\left(M\cup\:S\cup\:V\right)-L\right)$ but were not labeled as somatic or consensus calls, we removed them from the training set to achieve higher quality ground truth data during training time (Additional file 1: Table S5). 

#### Germline variant detection with DeepVariant

As the targeted sequencing confirmation focused on somatic variants, we aimed to increase the amount of ground truth labels for germline variants. Additional germline variants were added to the ground truth set using the following strategy. We ran DeepVariant v1.1.0 [59] on two normal replicates and combined the output by averaging over the quality score provided by DeepVariant. We then selected the variants with average quality scores above 45. DeepVariant predicted germline variant information was only used for training and validating the model and was not included in the evaluation step.

### TCGA-MC3 dataset and processing

The TCGA-MC3 dataset represents one of the most extensive resources for experimentally confirmed variants across diverse cancer types, including 22 cohorts, 3,639 patients, 3,655 tumor samples, 3,889 normal samples, and 177,806 calls (157,649 somatic and 227 germline variants, 19,906 loci with no mutation label) that were deep sequenced [47]. Despite confirming only a limited number of mutations per patient, this cohort enriched our training data with a wide spectrum of variants from different cancer entities, reflecting differences in tumor purity, clonality, and ploidy across cancer types. There was high variability in the mean number of confirmed calls across different cancer entities, ranging between [1, 271]. We used the reads around the variant positions with targeted deep sequencing labels for training VariantMedium deep learning models.

We first downloaded mc3.v0.2.8.CONTROLLED.maf.gz file from https://gdc.cancer.gov/about-data/publications/mc3-2017 to obtain labels in TCGA-MC3 dataset. We filtered this table such that validation_judgement_targeted field is filled, min_val_count_targeted is equal to or above 2, and validation_power_targeted is above 0.95. We normalized and left-aligned the variants using the tronflow-vcf-postprocessing pipeline [60]. The variants were then lifted over to GRCh38 using Picard LiftoverVcf (v2.21.2) ( https://broadinstitute.github.io/picard/ https://broadinstitute.github.io/picard/). This way, we obtained 157,799 validated and powered positive (somatic) labels and 19,775 unvalidated and powered negative (germline or not a mutation) labels with a median tumor VAF of 0.31 and an interquartile range (IQR) of 0.21. We then downloaded the corresponding matched tumor-normal BAM files and generated tensors for the targeted sites only.

### AML31 dataset and processing

We used AML31 dataset [46] to expand our training set with a hematological tumor type and with WGS based data. Notably, the platinum variant list presented in this study was generated from deep sequencing of 164,090 sites with a median coverage of 1,500x in the primary tumor, followed by extensive filtering and manual review. We downloaded the matched tumor-normal BAM files with IDs SRR2177258 and SRR2470200 for AML31 dataset. These files had high sequencing depth of coverage (312x), thus did not represent the clinical samples well.

Therefore, we downsampled the tumor and normal alignment files using DownsampleSam command from Picard v2.21.2 (https://broadinstitute.github.io/picard/) while keeping 20% of the reads. We preprocessed the BAM files using the tronflow-bam-preprocessing pipeline (v1.1.0), using the same procedure described for cell line BAM preprocessing. For label assignment, we first called candidate variants using Strelka2. We filtered the file by using SomaticEVS score > 4 and then joined Strelka2 candidates with the platinum SNV list. Those on the platinum list received positive (somatic) labels whereas the rest received negative (no mutation) labels. The 1,343 confirmed point mutations in the platinum set had median tumor VAF of 0.45 with IQR of 0.12.

### PCAWG-Pilot63 dataset and processing

The PCAWG study [24] provided WGS datasets across 38 tumor types with 2,658 matched tumor-normal sequences, 50 of which have gone through orthogonal targeted deep sequencing for somatic variant confirmation as part of the Pilot63 study. In the Pilot63 substudy, variants were called with seven somatic callers, and each sample was allocated 10,000 sites for sequencing, divided between 4,000 SNVs, 3,000 indels, and 3,000 structural variants. Importantly, the PCAWG consortium sequenced targets based on the concordance between different callers and allocated 30% of the targets for non-concordant calls.

We used the confirmed variants to evaluate the VariantMedium model with an independent dataset with extensive confirmation data by benchmarking variant callers on the targeted deep sequencing derived labels, computing caller performance using a concordance weighted benchmarking strategy, and assessing VariantMedium performance in different experimental conditions including varying sample purities, ploidies, variant VAFs, clonality classes and coverage at variant site. We utilized the whole genome sequencing data and variant status obtained via targeted deep sequencing of the 42 patients for which we obtained access. We excluded the samples with ICGC IDs DO220599, DO21115, DO36352, and DO36881 based on their graylisted or excluded status in the PCAWG sample sheet and additionally removed sample DO4557 due to missing VCF files. We assigned the label “somatic” to variants with “PASS” filter, the label “germline” to variants with “GERMLINE” filter, and the label “no mutation” to the rest of the filters (Additional file 1: Table S6). We removed variants with filter “LOWDEPTH” from the label set, as these did not have sufficient information to infer variant status. We downloaded the BAM files via gen3-client and preprocessed them using the tronflow-bam-preprocessing pipeline (v1.1.0) with known indels from GATK bundle and dbSNP. The resulting ground truth set contained data from 37 donors across 24 different cancer types, comprising of 94,044 somatic (71,866 SNVs, 22,178 indels), 6,367 germline (3,250 SNVs, 3,117 indels), and 78,574 no mutation (31,633 SNVs, 46,941 indels) labels with median tumor VAF of 0.2 and IQR of 0.25. The number of tested variants per-patient ranged from 421 to 6,742, with a median of 5,079 and an IQR of 1,042.

We downloaded the VCF files available for Broad, DFKZ, SVCP, and MuSE pipelines from ICGC via gen3-client. We ran NeuSomatic 0.2.1 on preprocessed BAM files using the provided reference genome, NeuSomatic_v0.1.4_standalone_SEQC-WGS-Spike.pth model, and BED files that include 300 bases before and after the deep sequenced variants. We ran VarNet v1.1.0 on preprocessed BAM files using the default options. We ran tronflow-strelka v0.2.1 which runs Strelka2 v2.9.10 with the default options on preprocessed BAMs. We ran tronflow-mutect2 v1.8.0 which runs GATK v4.2.6.1 on preprocessed BAMs, using gnomAD exomes v2.1.1 on GRCh37 reference as the input parameter for –gnomad, on preprocessed BAM files.

For the variant calling pipelines we used the GRCh37 reference genome. For the other callers, we used the downloaded VCF files.

We filtered the calls using bedtools [61] against RepeatMasker v3.2.7(20090120) [62], because the truth set was also filtered with this version of RepeatMasker [24]. We used VAFator v1.2.5 in tronflow-vcf-postprocessing pipeline to compute the VAF and coverage values that we use throughout the paper. We set the parameters –mapping-quality to 0 and –base-call-quality to 0 when running VAFator.

We obtained purity, ploidy, whole-genome doubling (WGD) status, and sex values from the ICGC database. Tumor mutation burden (TMB) was estimated by dividing the number of Mutect2 calls per patient by the total number of bases outside RepeatMasker regions. Since the performance values were not normally distributed, we used Spearman rank-order correlation to assess the association between precision, recall, F1 score vs. purity, ploidy, or TMB. We applied the Kolmogorov–Smirnov test to compare the distributions of these performance metrics between WGD and non-WGD samples and between male and female patients.

Clonality classes were obtained from Gerstung et al. [63] and the F1 scores over different clonality classes were computed on the subset of intersections between calls, labels, and variants with known clonality status.

### SEQC2-WES dataset and processing

The FDA-led SEQC2 consortium provided a standardized benchmarking dataset to systematically assess the accuracy and reproducibility of somatic variant calling across different experimental conditions, including library preparation protocols, sequencing platforms, and research centers [64]. To do this, SEQC2 consortium repeatedly sequenced the tumor cell line HCC1395 and its matching normal cell line HCC1395BL in different sequencing centers using different protocols and devices. By merging the output of these runs, they were able to achieve 2,000x WES coverage, and define somatic variants as follows: They called variants on 63 matched tumor-normal BAM files using different callers and defined confidence classes to classify a variant as somatic, based on the cross aligner and cross center reproducibility [65]. Based on these confidence classifications, they created a somatic variant truth set, which we use here for benchmarking variant callers using an additional well characterized independent WES dataset, and to differentiate the performances in low confidence vs. high confidence genomic regions. We assigned: “somatic” if a variant was labeled as HighConf or MedConf, we removed the variant if it was labeled as LowConf, and we assigned “no mutation” if it was in any call set of any variant caller and did not have HighConf/MedConf/LowConf label.

We downloaded the available aligned WES files, reference genome, exome and high confidence region BED files from http://ftp-trace.ncbi.nlm.nih.gov/ReferenceSamples/seqc. For BAM preprocessing, tronflow-bam-preprocessing pipeline was used with known indels from 1000 genomes, following SEQC2 consortium [64]. We used dbSNP v146 in bam-preprocessing pipeline and tronflow-bam-preprocessing v2.1.0.

We executed Mutect2, Strelka2, and VarNet using the tronflow-mutect2, tronflow-strelka pipelines, and VarNet docker image, respectively, applying the same software versions and parameters as used in the PCAWG analysis, apart from employing the GRCh38 reference genome and supplying all variant callers with the SEQC2 provided exome intervals file.

Fang et al. defined high-confidence genomic regions by running CallableLoci on each BAM file to identify areas of reliable read mapping [65]. Regions with low depth (< 10×), unusually high depth (> 8× the sample mean), low mapping quality (< 20), or low base quality (< 20) were excluded. They also removed sites marked as LowConf or Unclassified and three chromosomal segments showing normal loss of heterozygosity. The remaining regions, totaling 2.48 gigabase pairs, were included in high-confidence regions. We used bedtools subtract and bedtools intersect commands with -wa option to filter the VCF files [61] with SEQC2 consortium provided high confidence regions and to generate high confidence and low confidence subsets.

### Overview of VariantMedium workflow for somatic variant calling

**Fig. 1:**
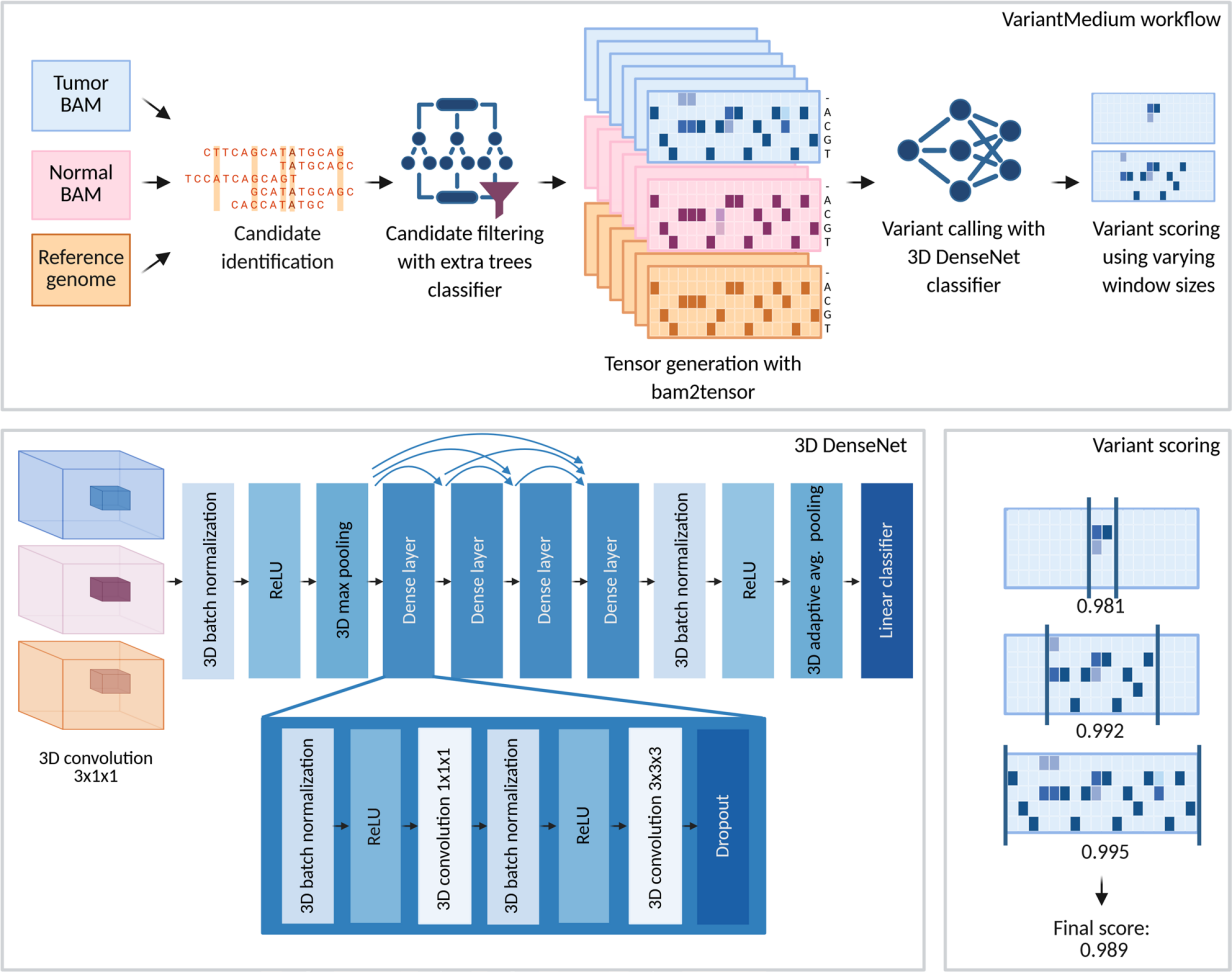
Overview of VariantMedium workflow for somatic variant calling. VariantMedium is a deep learning-based method that uses matched tumor-normal sequences, a reference genome, and a sensitive list of candidate variants to call somatic variants. First, candidates are filtered out using an ExtraTrees model. Next, 3D tensors are generated using the reads mapping to a defined range around the variant position. Then the tensors are input to a DenseNet with one block of four layers which outputs scores for three classes: no variant, somatic, germline. The classification is repeated for different window sizes around the candidate and final value is determined on the average of all scores. Created in BioRender. Muslu, Ö. [51] https://BioRender.com/5jh8wxn

To increase the accuracy of variant calling from matched tumor and normal WES or WGS data, we developed and trained the multi-model somatic variant caller VariantMedium. The workflow can be categorized into four modules: (1) candidate variant identification, (2) candidate variant filtering, (3) tensor generation, (4) variant calling (Fig. 1).

In the candidate variant identification module, we extract the candidate sites produced by Strelka2 prior to its variant calling stage to obtain a highly sensitive list of potential variants. In the candidate variant filtering module, we use an ExtraTrees classifier along with low-stringency filters to extract a list of candidate variants that preserve the sensitivity of the original list while removing majority of the candidates. In the tensor generation module, we convert the aligned reads around each candidate into tensors that provide structurally identical and cross-comparable matrices for tumor and normal sequences. In the variant calling module, we input the tensors to a 3D DenseNet [66] model with one block of four dense layers. The use of the DenseNet architecture avoids vanishing gradient problem and the use of 3D operators incorporates tumor, normal, and reference matrices already in the first few hidden layers, allowing for better comparison between them. Lastly, we compute the mean average score over tensors with different window sizes, which lets the final decision be guided by increased focus on variant position while still including surrounding bases in the decision. This step also enables variant calling in case of replicates, where we compute the final score by averaging over the scores originating from different technical replicates.

Overall, by optimizing candidate filtering, variant representation, model architecture and by training on experimentally confirmed sequencing data, we present a workflow that can predict somatic variants and rank them by their likelihood of being somatic.

### Candidate variant identification

To identify a highly sensitive (> 99%) candidate variant list for subsequent steps in our workflow, we used unfiltered Strelka2 (v2.9.9) candidates (where the FILTER field can be any value) generated with default Strelka2 parameters [21]. This list can include up to 3 million candidates depending on characteristics of the tumor (e.g. tumor mutational burden) and the parameters of sequencing (such as sequencing depth, breadth, or error rate).

### Candidate variant filtering

Filtering candidate variants with classical ML models is a strategy to remove easily distinguishable erroneous candidate variant calls, speeding up the variant calling process. For this purpose, we trained an ExtraTrees model on features calculated for the candidate positions using the tronflow-vcf-postprocessing pipeline v2.4.1 [60], which first normalizes and decomposes variants, then uses VAFator to compute features including VAF, sequencing depth, rank sum tests of variant position, mapping, and base qualities (Additional file 1: Table S7). 

To come up with the final model, we optimized number of trees, maximum tree depth, number of features to include when splitting the tree, and whether bootstrapping should be used in ExtraTrees models through a grid search on the validation set, using all features with parameter values shown in Additional file 1: Table S8. We then calculated feature importances and removed the features with feature importance values smaller than 0.01. We retrained the models and refit the hyperparameters within the same ranges. For differentiating between somatic variant candidates vs. negative examples, we selected a threshold that maximized F$\:{}_{\beta\:}$ score ($\:\beta\:$=5) on the validation set.

Lastly, we removed calls with (1) normal allele frequencies above 0.05 and normal coverage greater than 20, or (2) those supported by more than 2 normal reads, from further analysis to eliminate calls with normal evidence. We also removed calls with allele frequencies below 0.03 or fewer than 3 variant-supporting reads, as they had a very low probability of being somatic.

Python v3.9.12 [67] and Python libraries scikit-learn v1.0.2 [68], pandas v1.4.2 [69], and numpy v1.21.5 [70], joblib 1.2.0 [71] were used for this stage.

### Tensor generation

The goal of the tensor generation process is to represent mapped sequencing data at candidate variant loci as multi-dimensional numeric matrices (tensors), which can be used as input to deep learning models. We developed the Python library bam2tensor (https://github.com/TRON-Bioinformatics/bam2tensor) to generate input tensors for the VariantMedium models.

bam2tensor converts unstructured aligned read information in BAM files into structured sets of matrices without losing the information on the insertions by adding gaps to the reference sequence. Inputs to bam2tensor pipeline are a reference genome, a BAM file of tumor reads, a BAM file of normal reads, a candidate variants file in VCF or TSV format, and read length. The window size around the candidate variant can be adapted based on user requirements. The window size refers to the number of nucleotides before and after the variant start position. For the analyses presented here, we used a window size of 150, which captures entire reads, as the maximum read length in the datasets used here is 151.

The outputs of bam2tensor pipeline are 3D PyTorch arrays (tensors) of specified window size constructed around the candidate variants. The feature matrices contain information on the reference sequence, the start and end position of the variant aligned to the tensor (variant position in tensor), and the following information obtained from tumor and normal BAMs: matrix of VAFs of all loci within the window ($\:\alpha\:$), $\:\alpha\:$ weighted by base and mapping qualities, $\:\alpha\:$ separated for reads mapping to the forward and reverse strand ($\:\beta\:1$, $\:\beta\:2$), coverage values in $\:\beta\:1$ and $\:\beta\:2$, $\:\alpha\:$ weighted by edit distance of reads (to the reference genome), $\:\alpha\:$ filtered by unique mapping of the reads, matrix of variant allele frequencies of the variant supporting reads within the window that do not contain too many insertions/deletions and that do not support the variant at the beginnings or ends of reads, and their coverage (Additional file 2: Fig. S1). All matrices are represented as frequency matrices where each row corresponds to one of the four nucleotides or the gap character (-, A, C, G, T) and each column corresponds to a genomic position at or around the candidate variant. The resulting array size is 11 × 3 × 5 x (window size * 2 + 1), separated into 3 main channels for reference, tumor and normal sequences.

bam2tensor (v0.1.0) was used for this manuscript, which was written in Python v3.9.16 [67] using the packages bedtools v2.27.1 [61], samtools v1.15.1 [72], and the Python libraries numpy v1.24.2 [70], fire v0.5.0 [73], matplotlib v3.7.1 [74], pandas v1.5.3 [75], pybedtools v0.9.0 [76], pysam v0.20.0 [77], seaborn v0.12.2 [78], and torch v2.0.1 + cu117 [79].

### Data augmentation methods

We augmented the input data using three different techniques (Additional file 2: Fig. S2). During training, validation, and test stages, we used varying window size augmentation whereby both sides of all matrices are iteratively zeroed out, virtually making the window size smaller. This way, different window sizes are simulated, and the focus is directed towards the variant position, avoiding classifying other somatic or germline variants within the window while keeping the read information intact. We trained the models presented in this paper with the augmentation factor of 5.

We also employed tumor purity mixing and coverage downsampling techniques to augment the data points in the training set. In purity mix augmentation a proportion of randomly selected reads are removed from the tumor sample and replaced with randomly selected reads from the normal sample. In downsampling augmentation, a proportion of randomly selected reads are removed from both tumor and normal data. Purity mix and downsampling augmentation are implemented within the bam2tensor library. 

### 3D DenseNet and hyperparameter fitting

For variant calling, we used a DenseNet model with one block and four layers including 3D convolution, batch normalization, and max pooling layers, as our preliminary analysis on different DenseNet and residual network (ResNet) [80] architectures showed this as the outperforming architecture (Additional file 2: Fig. S3). In this model, the number of input channels is 8, and the number of initial features is 256, the growth rate is 32, and the bottleneck size is 4. The model has two classifiers, one for predicting variant status (somatic/germline/no mutation), another for predicting variant length class. For optimization, we used stochastic gradient descent and weighted cross entropy loss with the following weights: no mutation: 0.3, germline: 0.3, somatic: 0.4. We aimed to give balanced importance to each class at training time with this weight distribution to avoid optimizing the classifier for the majority class only. Additionally, we downsampled the majority class (no mutation) during training time such that the number of no mutation instances in one batch is roughly equal to the number of somatic variants during training.

We fine-tuned all models on the validation set to find the optimal augmentation rate for window size augmentation, purity augmentation, downsampling augmentation, learning rate, drop-out rate, number of layers, initial features, bottleneck value. For each model, we selected thresholds based on the validation sets, optimizing the F1 score, and used the same thresholds throughout our analysis. We compared different 3D DenseNet architectures by changing the hyperparameters such as number of initial features, number of blocks, and number of layers in each block, and decided on this architecture since it has the most consistent results in different validation sets when candidate variants with unknown labels are included (Additional file 2: Fig. S4). We did a final hyperparameter fit on learning rate, drop-out rate, augmentation mixes (Additional file 2: Fig. S5).

We optimized the 3D DenseNet SNV model using the training and validation sets described in Additional file 1: Table S2. We selected the model with the highest area under the precision–recall curve (AUPRC) on the validation set as the final model, since AUPRC is more informative than other metrics such as accuracy in imbalanced datasets (Additional file 2: Fig. S6).

We determined the final score of belonging to the somatic class by subtracting no mutation class score and germline class score from somatic class score. We then computed the mean average from different instances of the same variant site originating from window size data augmentation as the final score.

We evaluated the calibration of the predicted somatic probabilities by comparing predicted confidence values with empirical correctness frequencies using the targeted deep sequencing tested variants in 37 patients from PCAWG-Pilot63 dataset. For each caller, we binned predicted probabilities into 30 intervals and computed the fraction of true somatic variants in each bin. We plotted these empirical accuracies per bin against the mean predicted probability to obtain calibration curves. We also computed expected calibration error as the weighted average of the absolute differences between predicted and empirical accuracies across bins, with bin weights corresponding to the number of variants. All calibration analyses were performed at the variant level and restricted to SNVs.

We assessed the contribution of different feature groups to model predictions using a test-time ablation analysis using the targeted-deep-sequencing-tested variants in 37 patients from PCAWG-Pilot63 dataset. We organized all input channels into predefined groups: reference sequence, variant position in tensor, variant allele frequency, base and mapping quality scores, positive/negative strand VAF & coverage, depth of coverage, mapping metrics (edit distance, uniqueness), variant position in read, all matrices derived from tumor sample, and all matrices derived from normal sample. During evaluation, we ablated one feature group at a time by replacing all channels belonging to that group with mean-based substitutes derived from the remaining samples of the same category (reference, tumor, or normal). We then recomputed the classification scores under each ablation and measured the resulting drop in performance relative to the baseline model. Larger performance drops indicate feature groups with stronger influence on the model’s predictions.

The models were trained on Nvidia DGX-2. VariantMedium v1.1.0 was implemented in Python v3.9.16 [67] using CUDA v11.4 and Python libraries scikit-learn v1.2.2 [68], numpy v1.24.2 [70], fire v0.5.0 [73], pandas v1.5.3 [75] , and torch v2.0.1 + cu117 [79], and scipy v1.10.1 [81].

### Concordance weighted performance evaluation on PCAWG-Pilot63

To address biases arising from the targeted nature of the confirmation data and from unequal overlap between variant callers, we evaluated caller performance using a concordance-weighted benchmarking strategy as previously described by the ICGC/TCGA PCAWG Consortium [24]. We used the same method and implementation as described previously to benchmark gene fusion callers [82].

Variant calls from all callers were merged to define a unified call set. Each unique variant was assigned to a concordance bin based on the number of callers detecting that variant (e.g. concordance bin 1 for variants called by a single caller, bin k for variants called by k callers). Ground-truth labels from targeted deep sequencing were used to compute bin-specific performance measures, including sensitivity (recall) and precision.

Overall performance metrics were then calculated as weighted averages across concordance bins. Specifically, sensitivity was computed by weighting the bin-specific sensitivities by the total number of variants in each concordance bin across all callers. Precision was computed by weighting bin-specific precisions by the number of variants contributed by the caller under evaluation in each bin. The F1 score was derived from the resulting concordance-weighted sensitivity and precision estimates.

Concordance weighted performance comparison was implemented in R v4.1.0 [83] and R packages tidyverse v.1.3.2 [84], fs v1.5.2 [85], RColorBrewer [86], furrr v.0.3.1 [87], patchwork v.1.1.2 [88] was used.

### Cross sample stability

We quantified how strongly each caller’s performance varied across individuals by computing F1 scores separately for each sample and summarizing their variability at the caller level in 37 patients from PCAWG-Pilot63 dataset. For each caller, we calculated the median absolute deviation (MAD), IQR, mean F1 scores across samples, and we computed variance of sample-level F1 scores using a weighted formulation. In the weighted formulation, we aimed to account for differences in the number of labelled variants by weighing each sample by its number of labelled SNVs. This weighting accounts for the fact that samples with more variants provide more reliable performance estimates than samples with few variants. We report the weighted variance as the primary measure of cross-sample stability; lower values indicate callers whose performance depends less on the specific sample.

### Computational requirements

We quantified computational cost of VariantMedium v1.2.1 using Slurm accounting (sacct) by extracting elapsed time (Elapsed), peak resident memory (MaxRSS), and total CPU time (TotalCPU) for each workflow step. We report runtime using Elapsed, peak memory using MaxRSS, and compute effective CPU utilization as the average number of cores used, calculated as TotalCPU/Elapsed.

Across preprocessed SEQC2-WES BAM pairs, pipeline execution required a median of 2 h of compute time and up to 4 allocated CPU cores and 1 GPU, while average effective CPU utilization corresponded to approximately 2.5 cores and peak memory consumption reached 7.3 GB (Additional file 2: Fig. S7).

## Results

### High quality variant datasets with orthogonal confirmation data facilitate training of variant calling models

**Fig. 2:**
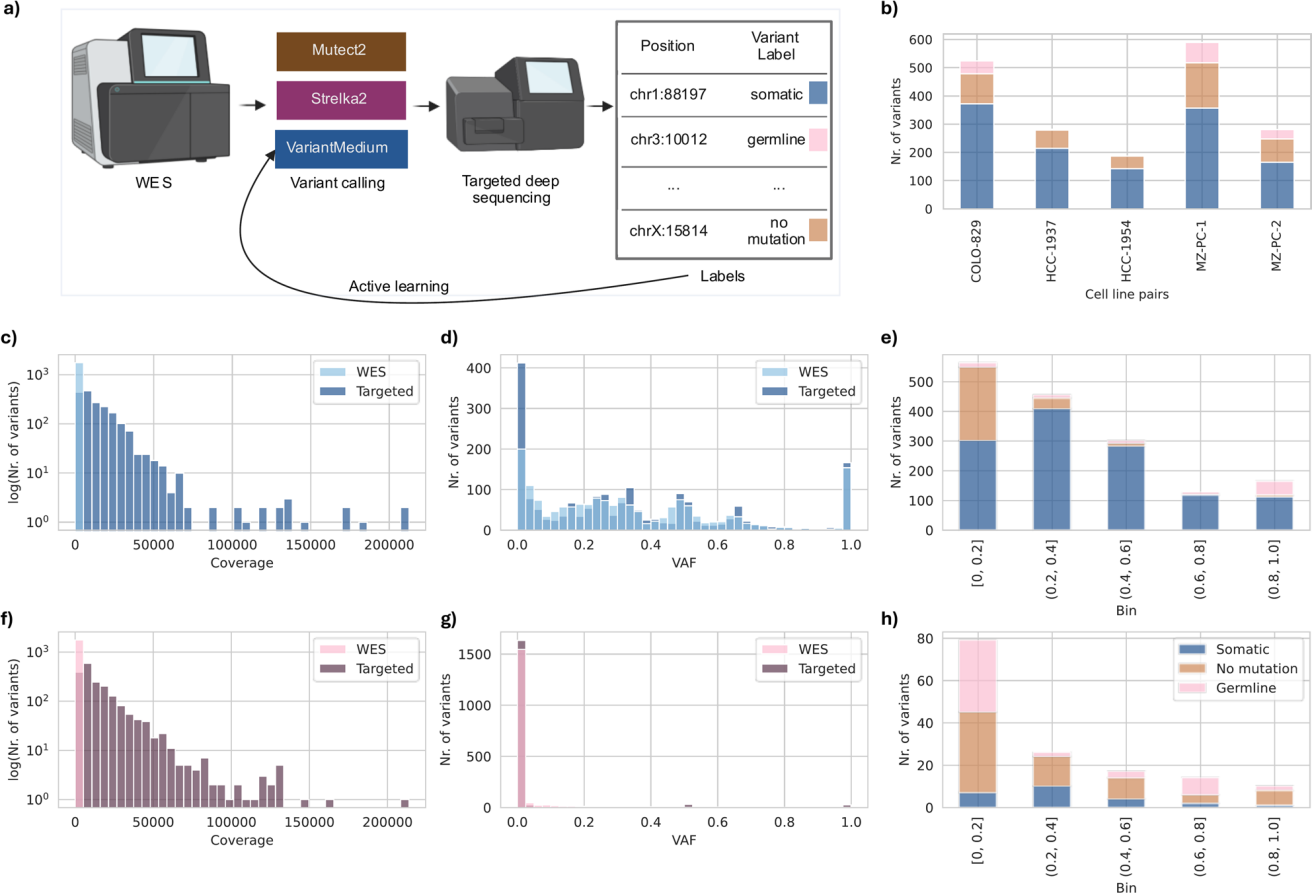
High quality variant datasets with orthogonal confirmation data facilitate training of variant calling models. **a** During the development phase of VariantMedium, we employed active learning by deep sequencing the calls from Mutect2, Strelka2, and VariantMedium in three batches and using them as input to VariantMedium in the next training round. **b** The distributions of deep sequenced variants into somatic/germline/no mutation classes as categorized by cell line. **c** Depth of coverage in tumor samples. **d** VAF in in tumor samples. **e** Number and classification of variants based on the VAF bin in SNVs. **f** Depth of coverage in normal samples. **g** VAF in normal samples. **h** Number and classification of variants based on the VAF bin in indels. Panel (**a**) created in BioRender. Muslu, Ö. [51] https://BioRender.com/2qtykiu

We generated WES and targeted deep-sequencing data of paired tumor and normal cell lines for the training of preliminary models. We used targeted sequencing derived labels to train our models, then called variants with VariantMedium, as well as two other somatic variant callers for further confirmation by targeted deep sequencing, and retrained VariantMedium with these labels, employing an active learning strategy (Fig. 2A).

First, for proof of concept and hyperparameter tuning, we sequenced whole exome libraries of 11 cell line pairs and confirmed the calls using targeted deep sequencing in five of these cell lines for training our preliminary models [51] (Additional file 1: Table S1). In a subset of three cell line pairs, we extensively confirmed all calls by Mutect2, Strelka2, and earlier versions of VariantMedium and retrained our models on these variants (Fig. 2A, Additional file 2: Fig. S8).

In total, we deep sequenced 1,663 SNV and 201 indel calls to get ground-truth labels in five cell lines. Altogether, 67% (1226 SNVs, 24 indels) of the deep sequenced variants were identified as somatic, 8% (91 SNVs, 64 indels) as germline, and 25% (346 SNVs, 113 indels) as no mutation (Fig. 2B, Additional file 1: Table S9). Next, we analyzed the distributions of coverage and VAF in WES and targeted sequencing data to ensure diversity in training data. WES coverage ranged from 2x to 1,000x with median coverages of 152x and 140x in tumor and normal samples, respectively (Fig. 2C, F). VAF values in WES had median of 0.285 in tumor samples and a median of 0 in normal samples; in targeted sequencing they had median VAF of 0.27 in tumor and 0 in normal samples (Fig. 2D, G). Notably, variants with tumor VAF below 0.1 had a lower rate of confirmed variants and consisted of 155 (37.1%) somatic variants, 37 (8.8%) germline variants, and 226 (54.1%) loci without any confirmed mutations (Fig. 2E, H). In the targeted deep sequencing confirmation data, the median coverage depth was 11,042x and 9,832x in tumor and normal samples, respectively. With this, we generated a novel variant ground truth dataset with comprehensive high-quality confirmations by targeted deep sequencing, which we use here for training.

### VariantMedium generalizes well to independent data with sequencing characteristics different than training data

A common problem of machine learning models is overfitting, thus it was crucial to demonstrate the performance of our somatic variant classification model on independent datasets. We evaluated the model’s performance on an extensive confirmation dataset published by the ICGC/TCGA PCAWG consortium, PCAWG-Pilot63 with 106,749 SNVs from 37 tumor patients with targeted deep sequencing confirmation data [24] (Methods, Additional file 1: Table S6). This strategy allowed us to assess the generalizability of our method on clinically relevant samples from diverse tumor types, spanning a range of tumor purities and technical characteristics distinct from the training and validation sets, including sequencing breadth (WES/WGS), sequencing depth (median 112× vs. 53×), and read length (51/76/100 bp vs. 101/151 bp) (Table 1, Additional file 1: Table S2). We applied VariantMedium to the matched tumor-normal WGS data for all downstream analyses.

First, we quantified how each step of the VariantMedium workflow affected the number of variants retained at each stage (Additional file 2: Fig. S9). The ExtraTrees classifier and downstream filters removed 91% of the 29,701,625 total candidates, reducing the set to 2,579,669, while retaining most confirmed somatic variants (from 88,116 to 84,059). The 3D DenseNet stage resulted in a modest additional reduction in confirmed somatic variants (to 62,924) and lowered the total number of candidates by 29% (to 1,831,965). Overall, the workflow eliminated most candidate variants while maintaining high retention of confirmed somatic variants.

Next, we evaluated the calibration of the 3D DenseNet model on loci confirmed by targeted deep sequencing using calibration curves with confidence intervals (Additional file 2: Fig. S10). The model showed mild underconfidence in low scoring variants but remained well calibrated in regions supported by sufficient data.

Next, we performed a test-time ablation study to assess feature importance by replacing correlating feature groups with mean-based substitutes computed on the rest of the features (Additional file 2: Fig. S11). Removing the variant position channels caused the largest drop in AUPRC (0.376), possibly because CNNs are shift-invariant and could detect neighboring somatic or germline events unless position cues anchor the model to the variant site. Ablating all tumor-derived matrices or all normal-derived matrices also produced substantial performance losses (tumor: 0.213, normal: 0.205). Other influential features included VAF (0.081), reference sequence (0.032), base and mapping quality scores (0.031), and depth of coverage (0.028).

We then assessed the overall detection accuracy of VariantMedium and benchmarked it against eight other somatic SNV callers by comparing precision, recall, and F1 score metrics on all deep sequenced variants. Specifically, we compared VariantMedium with calls from all PCAWG production pipelines (Broad, DKFZ, MUSE, and Sanger SVCP) [24], as well as newer versions of widely used variant callers Mutect2 [20] and Strelka2 [21], and two recently published deep learning–based methods, VarNet [43] and NeuSomatic [40] (Additional file 1: Table S10).

**Fig. 3:**
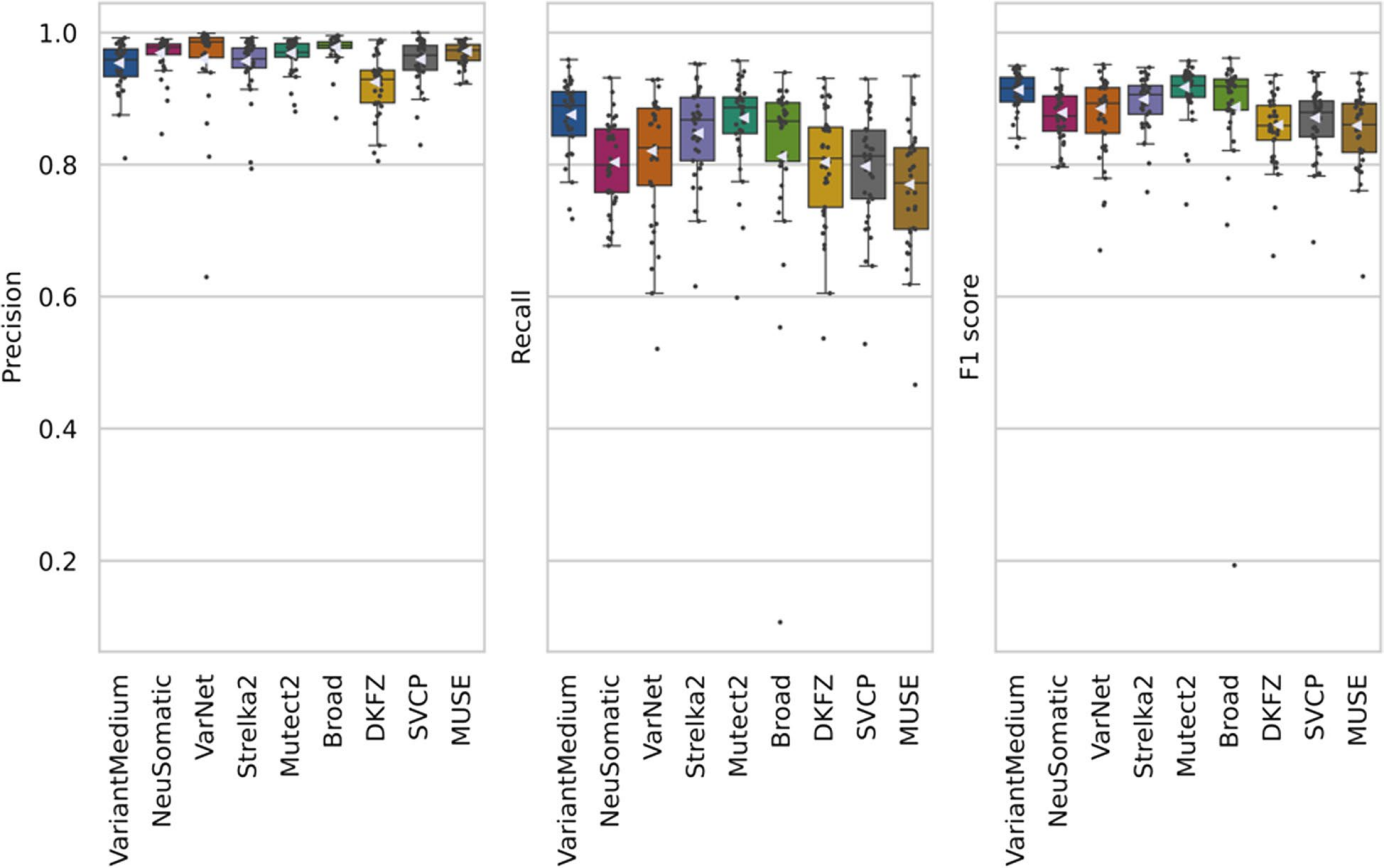
VariantMedium generalizes well to the independent dataset PCAWG-Pilot63 with sequencing characteristics different from the training data. SNV calling performance is shown as precision, recall, and F1 score of VariantMedium and other evaluated callers. Each dot depicts the performance of the given caller with respect to one patient, box plots indicate the distributions over the patients, and white triangles show the mean variant calling performance value for each caller over all variants

VariantMedium marked the highest sensitivity (VariantMedium: 0.876, Mutect2: 0.871, Strelka2: 0.848) and the second best F1-score with a small difference of 0.004 to the best performing method Mutect2, (VariantMedium: 0.913, Mutect2: 0.917, Strelka2: 0.899) (Fig. 3). Further, it showed the lowest weighted variance in F1 score distribution amongst 37 patients demonstrating low inter-sample variability (Additional file 1: Table S11). Considering the differences in coverage, read length, sequencing method, and center between the training and test sets, these results demonstrated that VariantMedium can generalize well to different datasets and provide a highly sensitive variant set with good precision.

Lastly, to understand the accuracy of calls on a patient-by-patient basis, we compared the differing calls between VariantMedium and Mutect2 pipelines in terms of the number of calls, F1 score, precision, and recall (Additional file 2: Fig. S12). VariantMedium specific calls produced higher F1 scores than Mutect2 in 25 patients, whereas Mutect2 specific calls produced higher F1 scores in 12 patients. When we limited this comparison to F1 score differences higher than 0.05, VariantMedium had higher F1 scores in four patients, whereas Mutect2 had higher F1 scores in two patients. Precision was generally higher for Mutect2, whereas the number of calls, recall, and F1 score varied between patients, indicating that one caller can be preferable than the other across different patients. Overall, the analysis with the PCAWG-Pilot63 targeted deep sequenced variants revealed high generalizability and accuracy of VariantMedium under conditions that differed from our training set.

### Concordance weighted benchmarking shows VariantMedium outperforms other callers in SNV detection accuracy

**Fig. 4:**
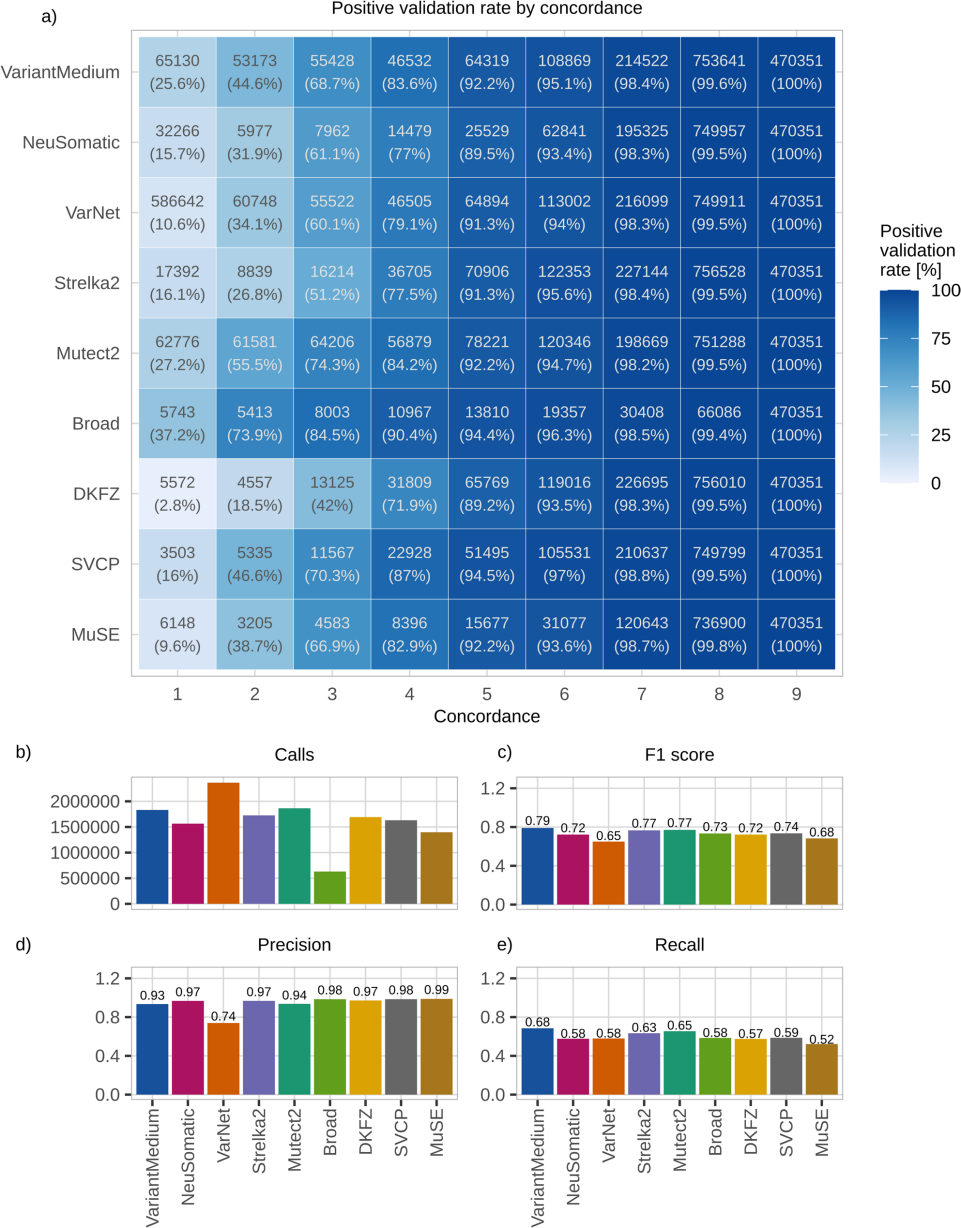
Concordance weighted performance comparison on PCAWG-Pilot63 dataset shows VariantMedium outperforms other callers in terms of sensitivity and F1 score. **a** Positive validation rate and the number of variants in concordance bins for all callers for SNVs. Concordance bin 1 shows the variants uniquely predicted by the given caller, concordance bin k shows the variants uniquely predicted by the given caller and k-1 callers. **b** The number of calls per sample. Concordance weighted F1 score (**c**), precision (**d**), and recall (**e**) over all variants. Concordance weighted performance comparison computes the precision, recall, and F1 score values in all concordance bins and then takes the weighted average w.r.t. the number of variants in each bin

In the analysis described above, we used ground truth data derived from targeted deep sequencing, which provides high-quality labels for loci of interest. However, because this ground truth was generated based on candidate call sets of other variant callers, the accuracy of variant calls that are not included before cannot be evaluated directly. Consequently, the performance analysis favors callers with high recall, since the additional calls, including potential false positives, are excluded from the evaluation. Additionally, it favors callers that were used to generate the targeted sequencing call set, as their calls are included in the ground truth, resulting in higher recall estimations. To overcome this limitation, we followed the concordance weighted benchmarking approach proposed by the ICGC/TCGA PCAWG Consortium [24, 82]. In this approach sensitivity and precision are calculated separately for groups of variants according to the number of callers (concordance bins) and weighted according to the fraction of calls with confirmation data in these groups. We applied this evaluation approach on PCAWG-Pilot63 dataset (Fig. 4).

First, we compared the number of SNV calls from all callers to assess whether the number of calls differ considerably between different callers (Fig. 4B). We observed that VarNet called the highest number of SNVs (2,363,674), followed by Mutect2 (1,864,317), VariantMedium (1,831,965), Strelka2 (1,726,432), DKFZ (1,692,904), SVCP (1,631,146), NeuSomatic (1,564,687), MuSE (1,396,980); whereas Broad Mutect (630,138) called notably less variants, which was mainly driven by two samples for which the other callers identified approximately 10-fold more variants.

Next, we analyzed the agreement among different callers by assigning variant calls into concordance bins, such that variants called only by a single caller would be in concordance bin 1 and variants called by k callers in concordance bin k. Concordance bin 1 with 785,172 variants was the most populated, followed by concordance bin 8 with 758,765 variants, indicating that whilst most callers agree on majority of the calls, there was a considerable fraction identified only by a single caller. Most calls in concordance bin 1 originated from VarNet calls (586,642) with a positive validation rate (PVR) of 10.6%. 65,130 calls in concordance bin 1 were identified by VariantMedium with a PVR of 25.6% (Fig. 4A), ranking third best PVR in this bin after Broad (37.2%) and Mutect2 (27.2%).

Finally, we used concordance-weighted performance calculation to compare the performances of different callers. VariantMedium had the best F1 score (0.79) with a margin of 0.02 to the next best callers, Mutect2 and Strelka2 (0.77), followed by SVCP and Broad pipelines (0.74, 0.73) (Fig. 4C). Interestingly, VarNet had lower precision compared to the previous unweighted analysis, leading to a lower F1 score (F1 = 0.65). VariantMedium had slightly lower precision compared to other callers (0.93 vs. [0.74, 0.99]) but its high recall made up the difference (0.68 vs. [0.52, 0.65]) (Fig. 4D, E). Concordance-weighted performance analysis showed that VariantMedium can predict variants with better accuracy than the other callers owing to its higher recall, in agreement with our previous analysis.

### VariantMedium shows consistent performance in different experimental conditions

Accurate variant calling is particularly challenging in clinical settings where tumor samples might contain a low fraction of tumor cells (low tumor purity) or harbor mutations present only in subclones (high tumor heterogeneity). Furthermore, the depth of sequencing coverage can vary greatly over the different genomic regions. Therefore, we evaluated VariantMedium performance in different VAF and coverage bins using the targeted sequenced loci in PCAWG-Pilot63 dataset. Next, we checked VariantMedium performance on different clonality classes. Lastly, we examined the statistical dependency between final F1 scores and potential confounding factors of estimated tumor purity, TMB, WGD, and patient sex.

**Fig. 5:**
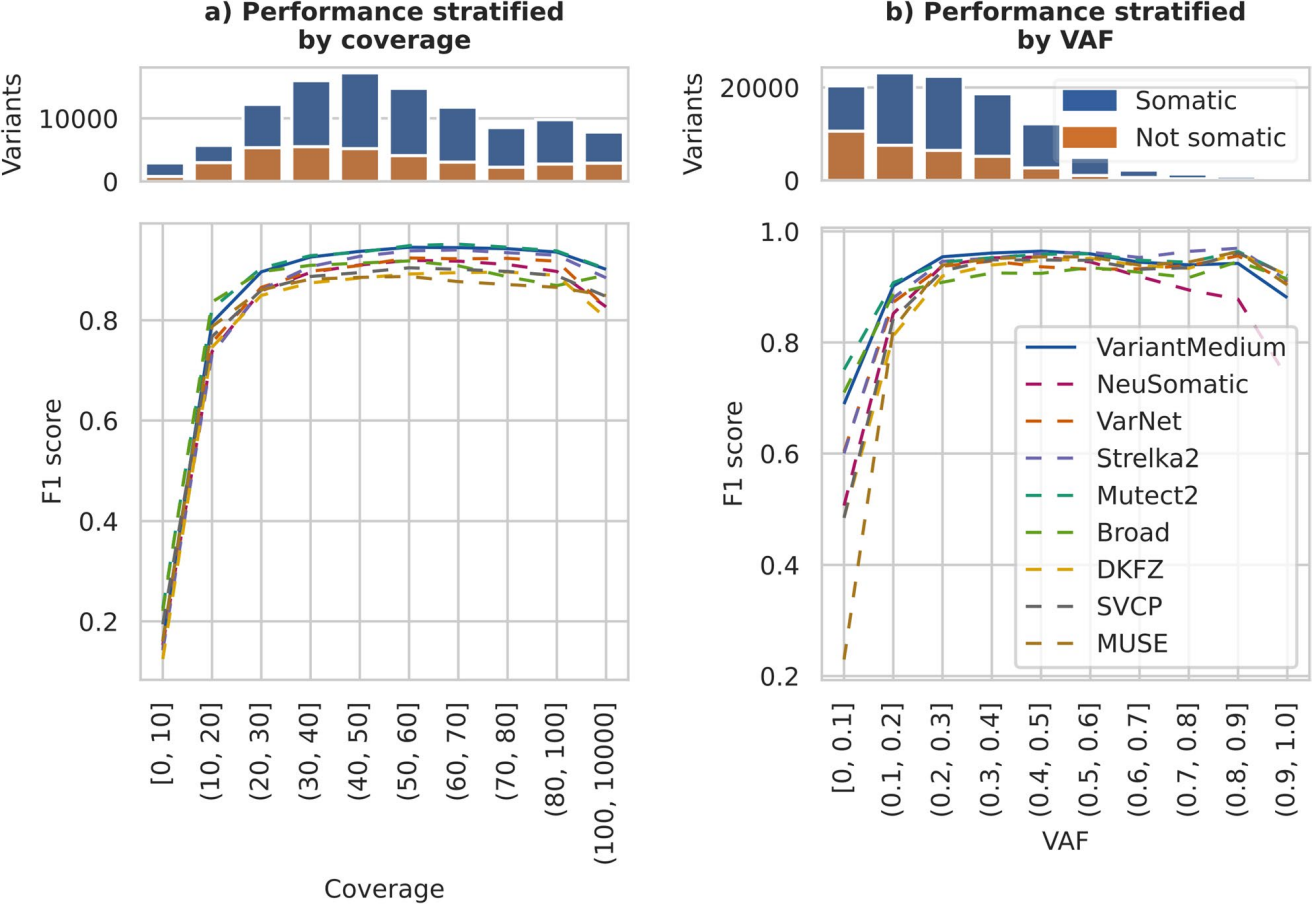
VariantMedium shows consistent performance in different VAF and coverage values in PCAWG-Pilot63 dataset. **a **The number of variants and F1 scores of different callers binned by coverage. The bar plots show the number of somatic and non-somatic variants for each bin and the line plots show the corresponding F1 scores of all callers in these bins. **b **The number of variants and F1 scores of different callers binned by variant allele frequency, illustrated the same way as in adium ranked among the top-performing callers across both coverage and VAF ranges.

Using the deep-sequenced variants, we first examined F1 scores across coverage ranges. VariantMedium showed stable F1 scores between 0.80 and 0.95 for sites with coverage above 10, and it outperformed all other callers except Mutect2 when coverage exceeded 20 (VariantMedium: 0.9–0.95; Broad Mutect: 0.9–0.92; Mutect2: 0.9–0.95) (Fig. 5A). VariantMedium also maintained high F1 scores across VAF ranges, with a minimum of 0.69 in the 0–0.1 interval, following Mutect2 and Broad Mutect, which reached 0.75 and 0.71, respectively (Fig. 5B). Overall, VariantMedium ranked among the top-performing callers across both coverage and VAF ranges.

Next, we assessed F1 scores across clonality classes defined by the PCAWG Evolution & Heterogeneity Working Group and the PCAWG Consortium [63] (Additional file 2: Fig. S13). VariantMedium, Broad Mutect, and Mutect2 showed the most reliable performance across clonality classes consistently ranking among top three callers in all four classes. VariantMedium achieved the highest F1 score in the group “clonal [NA]” (0.967), MuSE in the group “clonal [early]” (0.954), Broad Mutect in the group “clonal [late]” (0.967), and Mutect2 in the group “subclonal” (0.941).

Lastly, we investigated whether there is any statistically significant relationship between the variant calling accuracy and tumor purity, tumor ploidy, WGD, TMB or patient sex using Spearman rank-order correlation or the Kolmogorov–Smirnov test (Additional file 1: Table S12, Additional file 2: Fig. S14). We did not find any significant associations except for TMB, where a positive correlation with precision (Spearman ρ = 0.339) and negative correlations with recall (Spearman ρ=-0.636) and F1 score (Spearman ρ=-0.632) were observed.

In conclusion, we showed that VariantMedium confidently calls variants at typical coverage levels above 20 and achieves detection performances comparable to other callers on low-VAF variants. It performed consistently amongst different clonality classes, including the subclonal variants and showed no dependence on tumor purity, ploidy, WGD status, or patient sex in the tested samples.

### Deep learning based variant callers outperform classical methods in low confidence genomic regions

As the final part of our analysis, we evaluated VariantMedium on an additional independent dataset, SEQC2 [64, 65, 89], to further assess its generalizability and applicability across different genomic regions. While this dataset has been derived from only one breast cancer cell line HCC1395 and its matching B lymphocytes cell line HCC1395BL, it was sequenced thoroughly with > 2000x WES coverage using different platforms and centers [64, 65, 89]. This level of sequencing coverage in a cell line suggests that nearly all exon variants in this sample pair were identified, therefore we consider the resulting call set as complete. Using this call set, we benchmarked VariantMedium, VarNet, Mutect2, and Strelka2, but excluded NeuSomatic since the NeuSomatic model we used was trained partly on SEQC2, and some labels in SEQC2 were derived from consensus NeuSomatic calls directly. We used 12 WES pairs from different sequencing centers with varying coverage depth to assess VariantMedium’s performance in an independent WES dataset, which showed varying characteristics to our training set (Table 1).

**Fig. 6:**
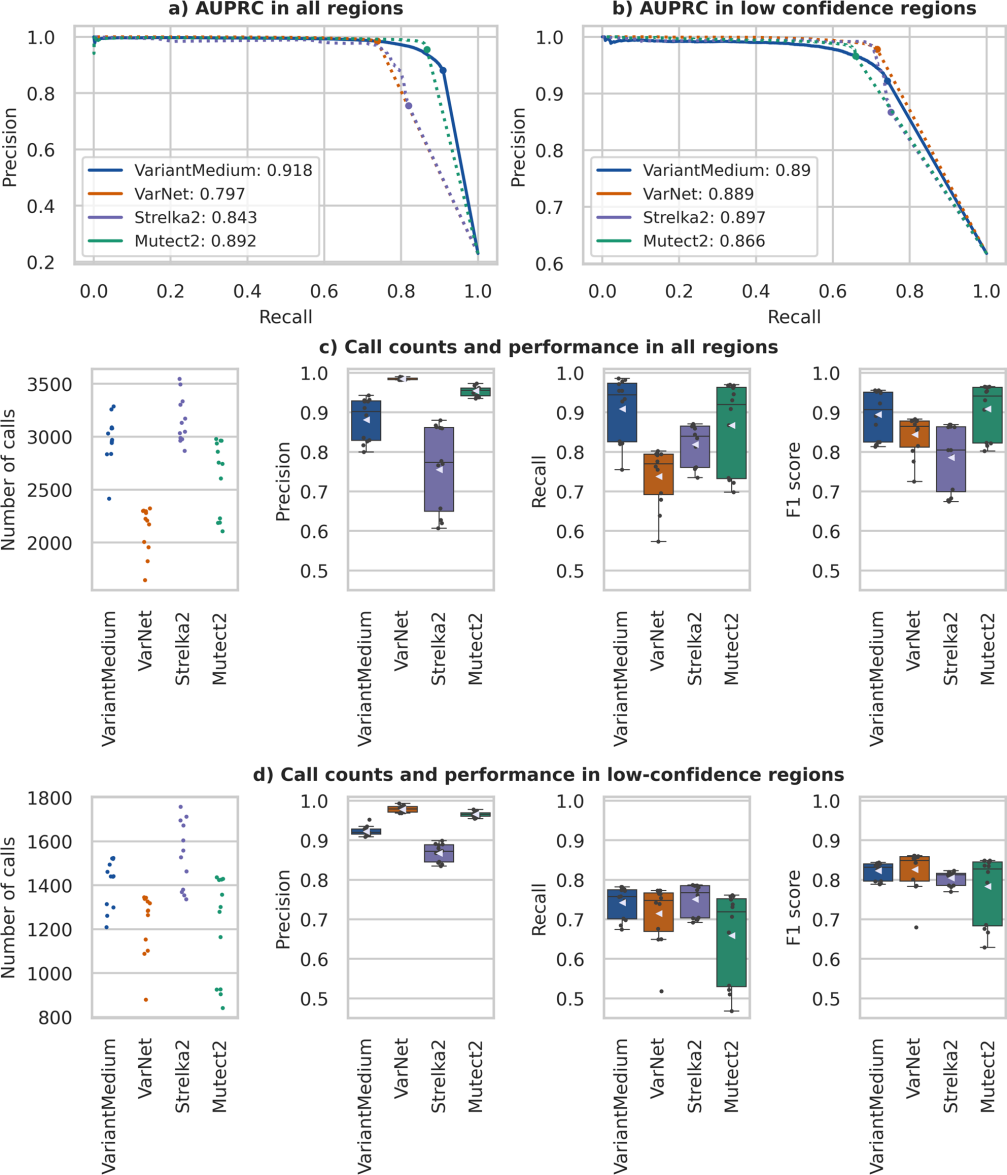
Deep learning based variant callers have higher F1 scores in high complexity genomic regions on SEQC2 WES dataset**.** Precision-recall curves and AUPRC values for (**a**) all regions and (**b**) low confidence regions. Dots indicate the F1 scores of final variant call set for each caller. Number of calls per sample, precision, recall, and F1 scores of benchmarked callers in (**c**) all regions and (**d**) low confidence regions. In boxplots each dot depicts the performance (precision, recall, or f1 score) of the given caller with respect to one sample, box plots show the score distribution over different samples, and the white triangles show the mean variant calling performance value for each caller over all variants

We first assessed the performances of the four callers in all exome regions in terms of AUPRC, precision, recall, and F1 score (Additional file 1: Table S13). VariantMedium achieved the highest AUPRC (VariantMedium: 0.918, Mutect2: 0.892, Strelka2: 0.843), highest sensitivity (VariantMedium: 0.909, VarNet: 0.738, Strelka2: 0.819, Mutect2: 0.867) and second highest F1 score after Mutect2, mirroring the results in PCAWG-Pilot63 dataset (VariantMedium: 0.895, VarNet: 0.844, Strelka2: 0.786, Mutect2: 0.909) (Fig. 6A, C). Furthermore, the F1 score differences between different sample pairs were smaller in VariantMedium ([0.813, 0.956]), compared to all others (Mutect2 :[0.802, 0.965], Strelka2: [0.674, 0.869], VarNet: [0.725, 0.883]).

The SEQC2 consortium additionally defined high confidence genomic regions where short read mapping is not hindered by high GC content or low complexity and where conventional variant callers tend to perform reliably [65]. Although variant calls in these regions are typically more robust, the remaining regions still contain biologically important loci, including genes such as *HLA*. Therefore, we next explored the ability of benchmarked callers to call outside these high-confidence regions, which we named here low confidence regions. Here, Strelka2 showed the highest AUPRC (0.897) followed by VariantMedium (0.89) (Fig. 6B), however this was not well reflected in Strelka2’s F1-score of 0.805, ranking third after VarNet (0.826) and VariantMedium (0.823) (Fig. 6D). Mutect2 ranked last in both F1 score and AUPRC in low confidence regions and had the highest variability in performance across different sample pairs (Fig. 6D). Although VarNet had slightly higher F1 scores than VariantMedium in this region, VariantMedium showed less variability across samples. The results indicate that while VarNet may be preferred for higher precision, VariantMedium again offers higher sensitivity. Overall, deep learning–based callers performed best in low-confidence genomic regions.

In summary, VariantMedium demonstrated strong generalizability in the SEQC2 dataset and delivered favorable performance in low-confidence regions of the exome.

## Discussion

Although diverse approaches have been developed for somatic variant calling, detecting somatic mutations in complex clinical samples with noisy data still has unresolved challenges, especially when the sequencing coverage or tumor purity is low. Advances in deep learning in the past years show marked improvements, yet multiple bias sources and technical challenges still need to be considered when training and validating such models. Specifically, limited generalizability of existing approaches revealed the need for a robust methodology which can handle experimental error sources across a variety of sequencing strategies for SNV detection across different tumors and cell types.

Here, we presented a novel somatic variant caller built on a multi-model architecture using an ExtraTrees classifier followed by a 3D DenseNet trained on matched tumor-normal exome sequencing of cell lines and tumor samples with orthogonal targeted deep sequencing data, evaluated on two independent datasets. Key advances of VariantMedium over state-of-the-art callers are the use of 3D convolutions to better model complex sequencing data by capturing tumor and normal information together, employing DenseNets to capture local and global genomic context without losing the input structure, training and evaluation on real datasets that represent diverse technical and biological biases, using ExtraTrees models for initial candidate filtering, and incorporation of multiple data augmentation approaches for training, especially the varying window size augmentation. By employing these methods, we achieved exceptional and generalizable variant calling performance in SNV calling, particularly in terms of sensitivity, while attaining similar/better F1 scores when compared to other callers.

We measured the performance of our model in independent whole genome sequencing dataset from the PCAWG-Pilot63 study on more than 106,000 orthogonally tested variants and demonstrated that our SNV model generalizes well and outperforms all other pipelines in concordance-weighted F1 score calculation as a result of higher recall values. Notably, VariantMedium also outperformed the recently published deep learning based somatic variant callers NeuSomatic [40] and VarNet [43]. Presumably, NeuSomatic could benefit from experimentally sourced and more variable training data. VarNet has the limitations of generating tensors for a coverage depth of maximally 100 reads and the use of consensus variants as the ground truth, which likely hindered its performance by limiting sensitivity. In conclusion, the PCAWG test set analysis demonstrated that VariantMedium outperform state-of-the-art variant detection pipelines despite being trained on datasets with differing data characteristics to this dataset.

We further assessed our approach in more than 12,000 variants from 12 WES pairs shared by SEQC2 consortium and showed the generalizability and consistently high sensitivity of our model. Additionally, we demonstrated the favorable performance of deep learning based approaches, VariantMedium and VarNet, in low confidence regions with subpar short read mapping.

Similar to what was described for VarNet, the indel calling performance of VariantMedium is limited compared to other callers (Additional file 2: Fig. S15). This might be explained by the limitation of current datasets and deep learning-based methods to model the more complex and noisier indel variant space. Future studies may focus on more indel-specific algorithms and validation of more indel candidates for more robust ML models.

## Conclusions

We provide VariantMedium as a somatic variant caller together with the source code and all pre-trained prediction models under an open-source license on GitHub. Users can adjust the trade-off between precision and sensitivity by raising or lowering the default threshold applied to the 3D DenseNet score (Additional file 2: Fig. S16). While we demonstrated high generalizability of the model, the machine learning based implementation allows retraining for more specific use cases, such as data from FFPE samples or long-read sequencing technologies with different error profiles than the sequencing technology from Illumina [90, 91]. Overall, we provide a highly accurate and generalizable method to detect somatic SNVs from diverse WES and WGS data, which will allow better characterization of tumor genomes and ultimately contribute to improving personalized cancer therapies.

## Supplementary Information

Additional file 1. Supplementary Tables (Table S1-S13).

Additional file 2. Supplementary Figures (Fig. S1-S16).

## Data Availability

The whole-exome sequence data from cell lines COLO-829, HCC-1937, HCC-1954 have been deposited in the European Genome-phenome Archive (EGA accession EGAS50000001661). The AML31 dataset used for the analyses described in this manuscript were obtained from dbGaP through dbGaP accession number phs000159.v13.p5. Sequencing data for the PCAWG-Pilot63 study were provided by the TCGA and data for both datasets were obtained through dbGaP accession phs000178.v11.p8.The SEQC2 dataset was downloaded from https://ftp-trace.ncbi.nlm.nih.gov/ReferenceSamples/seqc/Somatic_Mutation_WG/. The source code and documentation of VariantMedium is available under an open source on GitHub: https://github.com/TRON-Bioinformatics/VariantMedium [92]. The code is written mainly in Python, and pipeline is established using Nextflow and Shellscript. The dependencies for running the pipeline are nextflow >= 25.10.4, conda >= 26.1.1 and CUDA 13.2 (optional in beta mode).  The bam2tensor python package is available under an MIT license here:https://github.com/TRON-Bioinformatics/bam2tensor and requires Python 3.9+.

## Abbreviations

AUPRC: Area under the precision-recall curve
DenseNet: Densely connected convolutional network
EGA: European Genome-phenome Archive
ExtraTrees: Extremely randomized trees
IQR: Interquartile range
ML: Machine learning
MAD: Median absolute deviation
PVR: Positive validation rate
ResNet: Residual network
SNV: Somatic single nucleotide variant
TMB: Tumor mutation burden
VAF: Variant allele frequency
WES: Whole exome sequencing
WGD: Whole-genome doubling
WGS: Whole genome sequencing
