# Supplementary material for: VariantMedium: sensitive and generalizable somatic point mutation calling with 3D DenseNets trained and evaluated on experimental data
Source: Genome Med. 2026 Jun 19;18:89. doi: 10.1186/s13073-026-01675-1 (PMC13285312; doi:10.1186/s13073-026-01675-1)
Supplement: Supplementary file 2 — Additional file 2. Supplementary Figures (Fig. S1-S16). [file 13073_2026_1675_MOESM2_ESM.pdf]

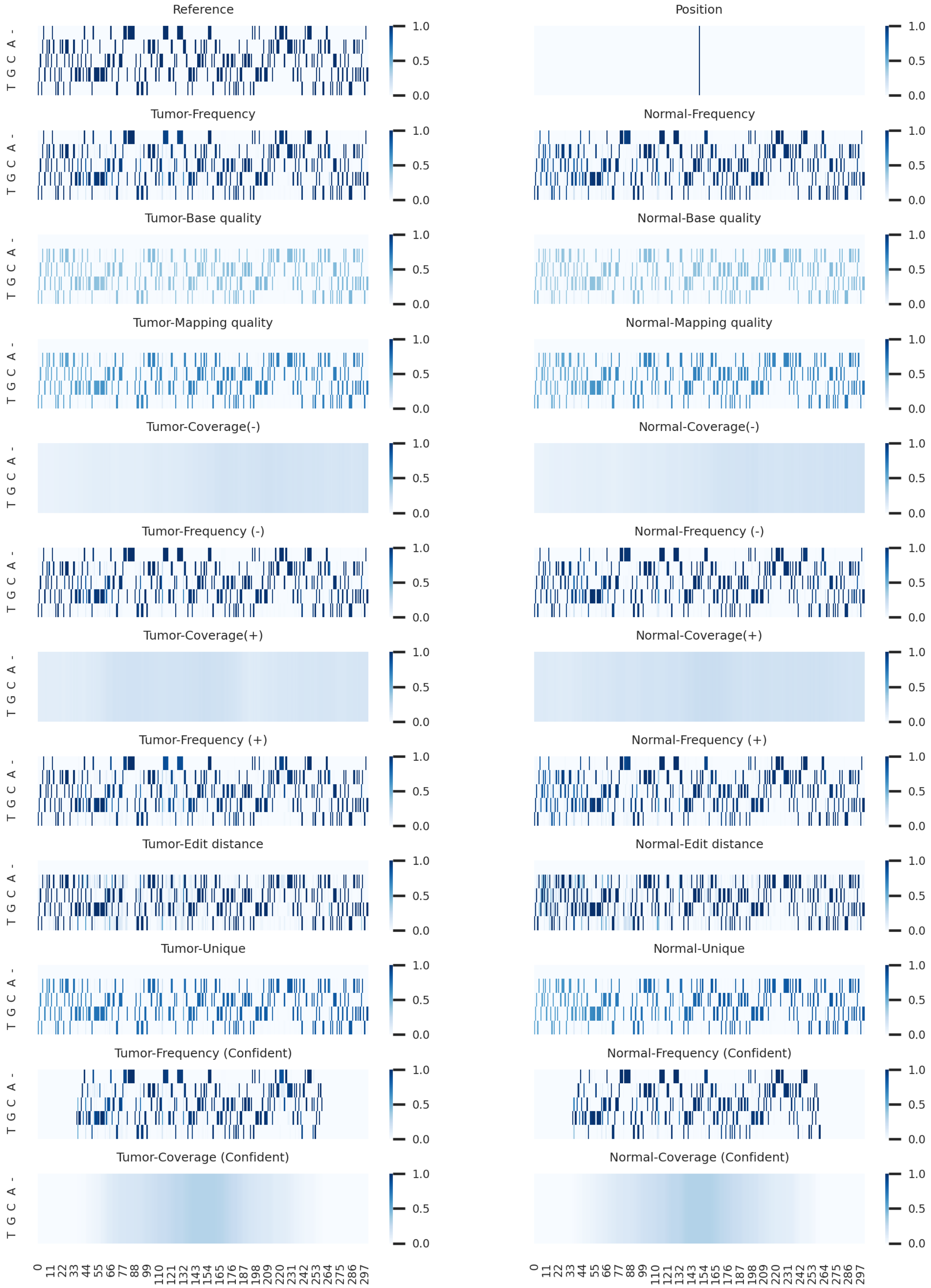

Figure S1: Example of a tensor generated by bam2tensor library with a window size of 150 (read length: 50). Here, each box illustrates one feature matrix obtained from reference, tumor, or normal sequences. Rows represent the four bases and the gap character (-, A, C, G, T), and columns represent the encoded information for the relative position in the genome, with variant start position located in the middle of the tensor.

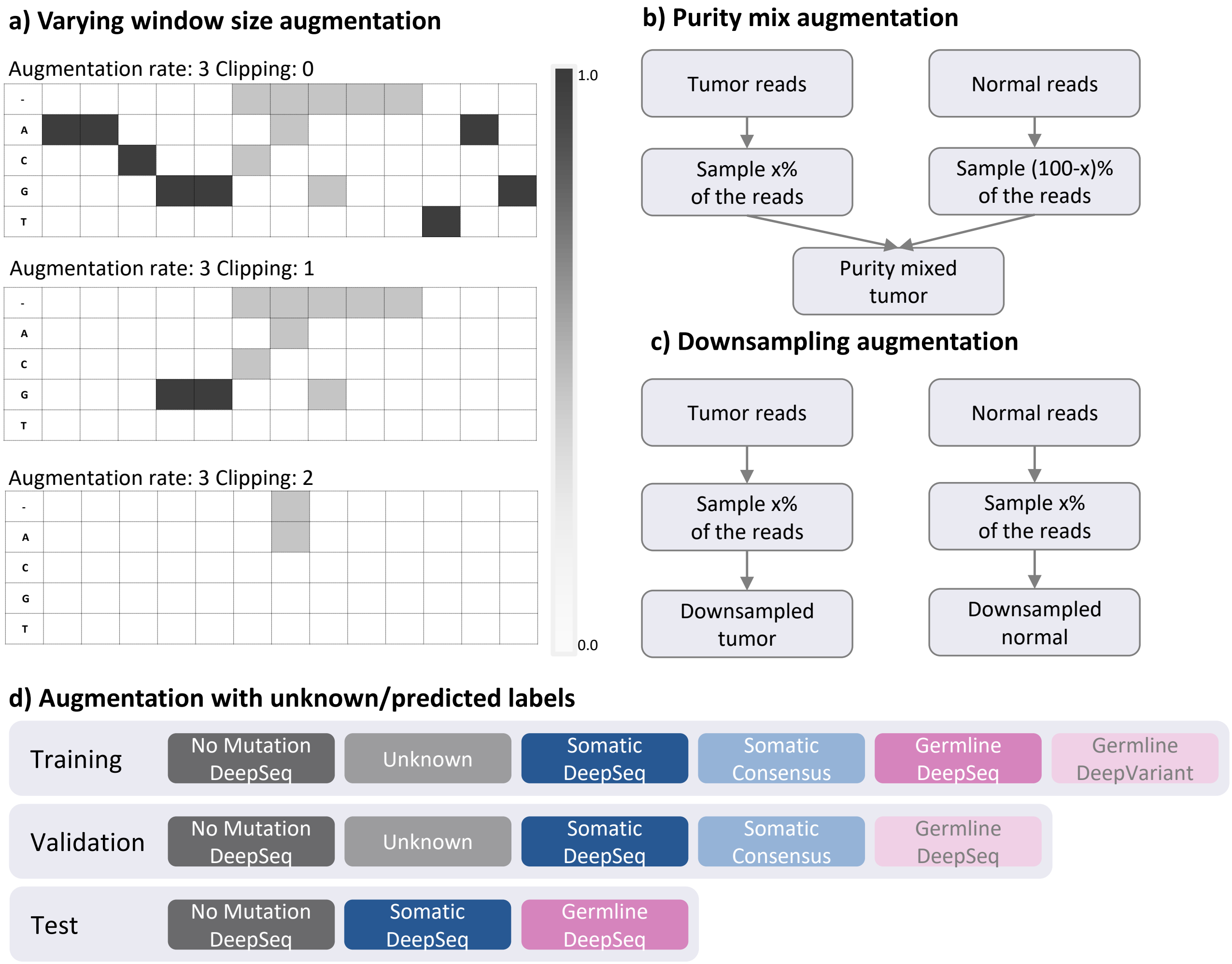

Figure S2: Data-augmentation approaches in VariantMedium. (a) Varying window size augmentation takes the original tensor and zeroes out the cells on the sides to mimic a smaller window size. (b) Purity-mix augmentation simulates lower tumor purities by mixing tumor and normal reads in user-defined ratios. (c) Downsampling augmentation randomly selects a user-defined ratio of reads and removes them. (d) Usage of unknown/predicted labels increases the size of labelled cell line data by assigning labels to variants even when targeted deep sequencing data is missing: germline variants are augmented by including DeepVariant predictions, somatic variants by including consensus calls, and negative data points by assigning a “no mutation” label to unknown examples.

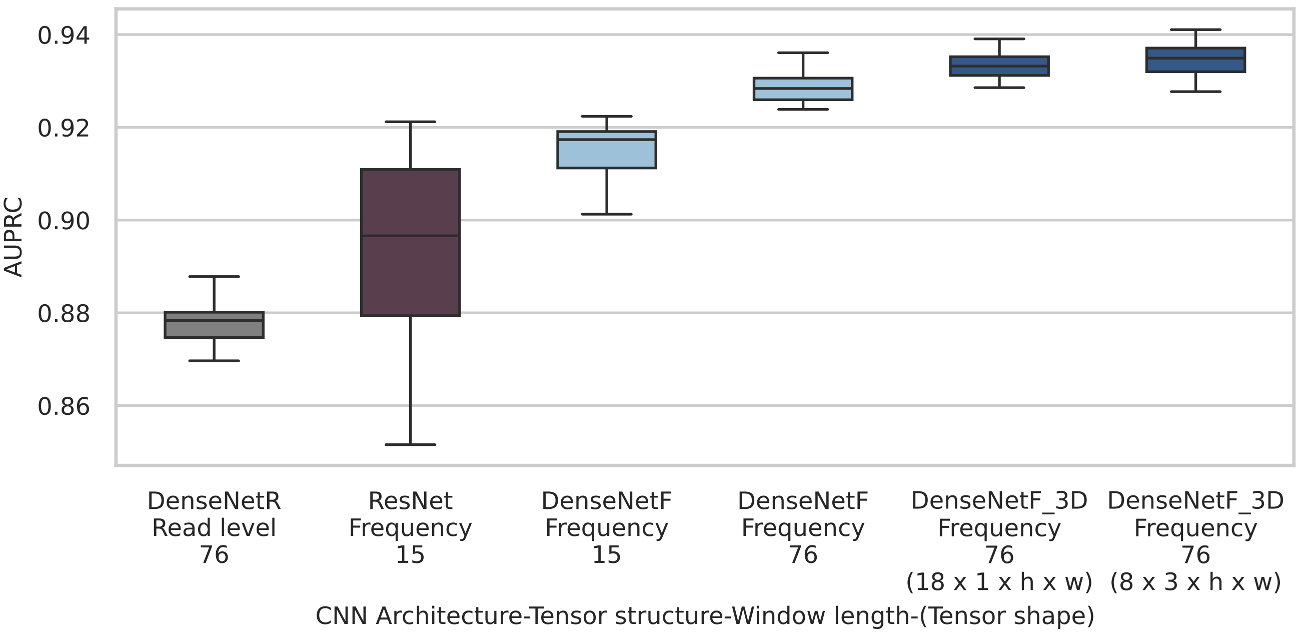

Figure S3: Preliminary analysis on neural network architecture, tensor encoding, window size, and (where applicable) tensor shape. Using preliminary cell line data from first round of targeted deep sequencing, we compared the performance using different tensor encodings and neural network architectures. Each box shows the performance distribution of models trained using different learning rates between 0.007 and 0.017. ResNet denotes a residual CNN with a conv-BN-pool input layer, followed by four residual blocks with increasing dilation. DenseNetR denotes an initial conv–BN–ReLU–pool stem, followed by four dense blocks with 6, 12, 24, and 16 layers. Transition layers include 1×1 convolutions and average pooling. DenseNetF referes to a 2D DenseNet variant with a conv–BN–ReLU–pool stem (kernel 1×3), followed by two dense blocks with six layers each, using a growth rate of 16 and bottleneck factor 4. Transition layers include 1×1 convs and 2D average pooling. DenseNetF_3D is a 3D DenseNet with a conv–BN–ReLU–pool stem, followed by a dense block of 4 layers using a growth rate of 16 and bottleneck factor 4. Read-level tensors are those that contain information on singular reads per each row, and frequency tensors are those that concatenate the values in the positions of interest and thhe window around it, as explained in Methods. Window size refers to the number of chromosome positions included before and after variant position. Tensor shape is given and compared for the 3D DenseNets.

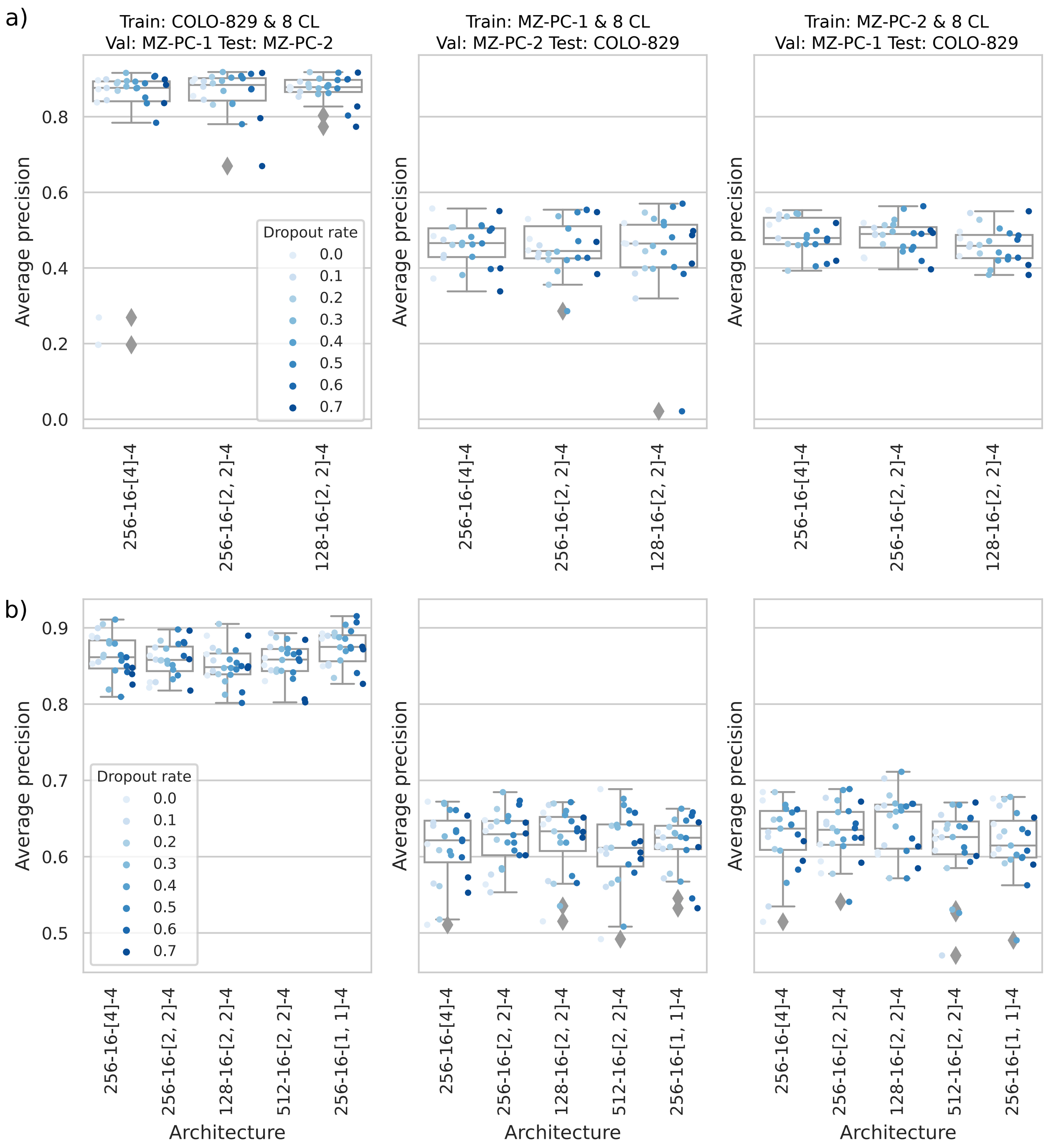

Figure S4: Preliminary 3D DenseNet architecture comparison. We compared several 3D DenseNet architectures for SNV (a) and INDEL models (b) after the second round of targeted deep sequencing. We trained model variants that differed in initial feature count, growth rate, block configuration, and dropout settings, and we evaluated their performance on validation sets that also included candidates with unknown labels. We summarized the resulting AUPRC distributions with box plots. Each point represents AUPRC value from one model trained with dropout values between 0 and 0.7 x learning rates of 0.1, 0.01, or 0.001. All models used an augmentation set consisting of purity mixtures (0.75, 0.5, 0.25), downsampled tensors (0.9), and combined downsampled and purity-mixed tensors (0.8)

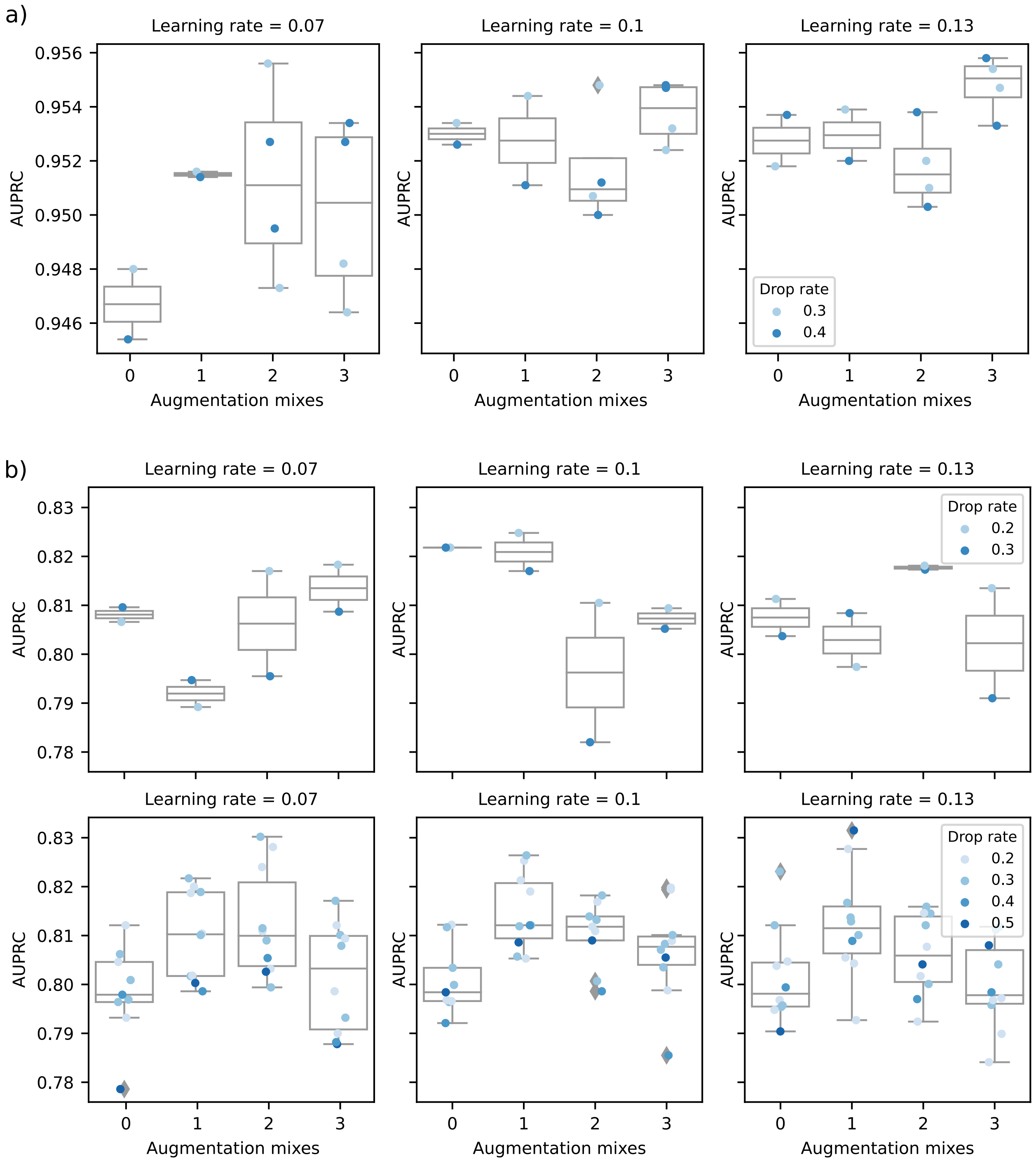

Figure S5: Final hyperparameter optimization. We performed a final hyperparameter optimization after the third round of deep sequencing for SNV (a) and INDEL models (b) on the finalized validation set (Supplementary Table 2). In addition to architectural choices, we optimized learning rate, dropout rate, augmentation mixes, and, for INDELs, the block configuration. We fully trained all model variants and summarized their performance with box plots showing the AUPRC distributions; each point represents the AUPRC of one model on the validation set. The x-axis in each plot indicates the augmentation mixtures used, with the number of applied augmentation sets increasing from left to right. In panel (b), the top row corresponds to a block configuration with one block of four layers, and the bottom row corresponds to two blocks with one layer each.

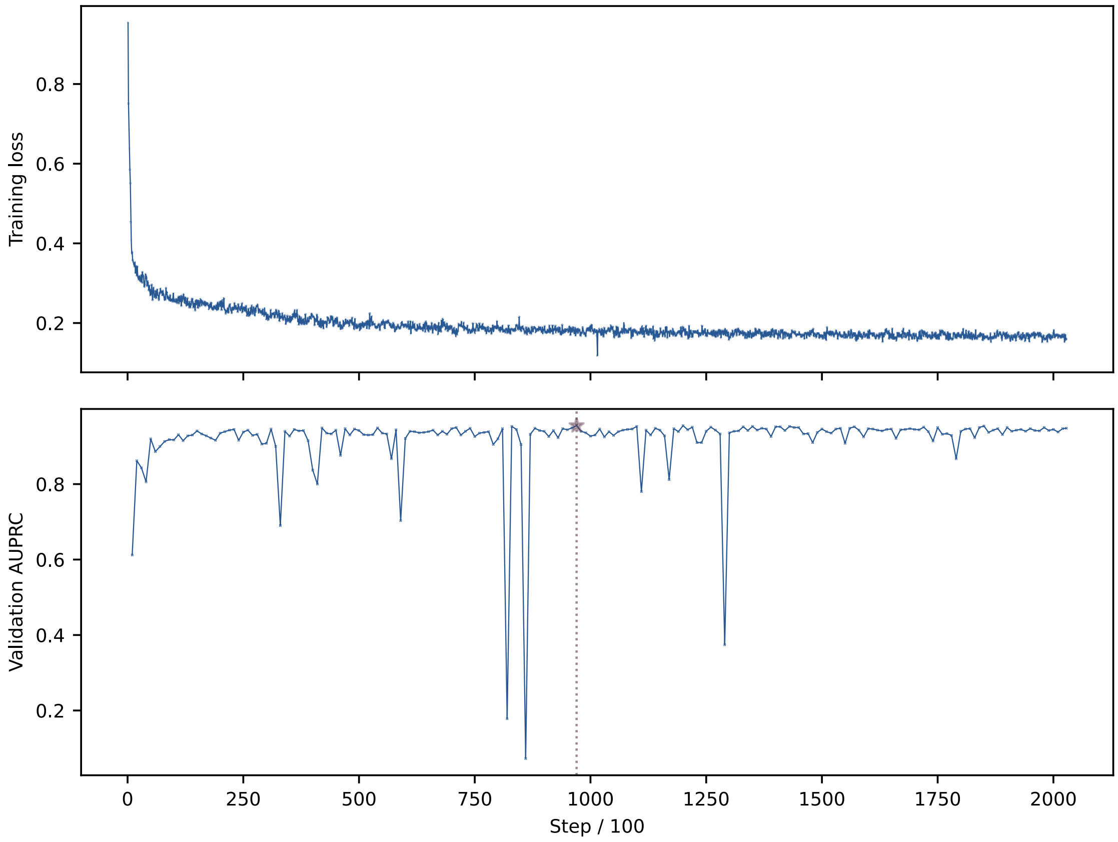

Figure S6: Parameter fitting in the proposed 3D DenseNet SNV model using the training and validation sets outlined in Supplementary Table 2. The top row shows the training loss computed using a weighted cross-entropy objective (weights: somatic = 0.4, germline = 0.3, no-mutation = 0.3). Because of the strong class imbalance with a high proportion of negatives, we used validation AUPRC as the primary evaluation metric, shown in the bottom row. The star and vertical dashed line in the bottom plot indicate the model selected for the final evaluation.

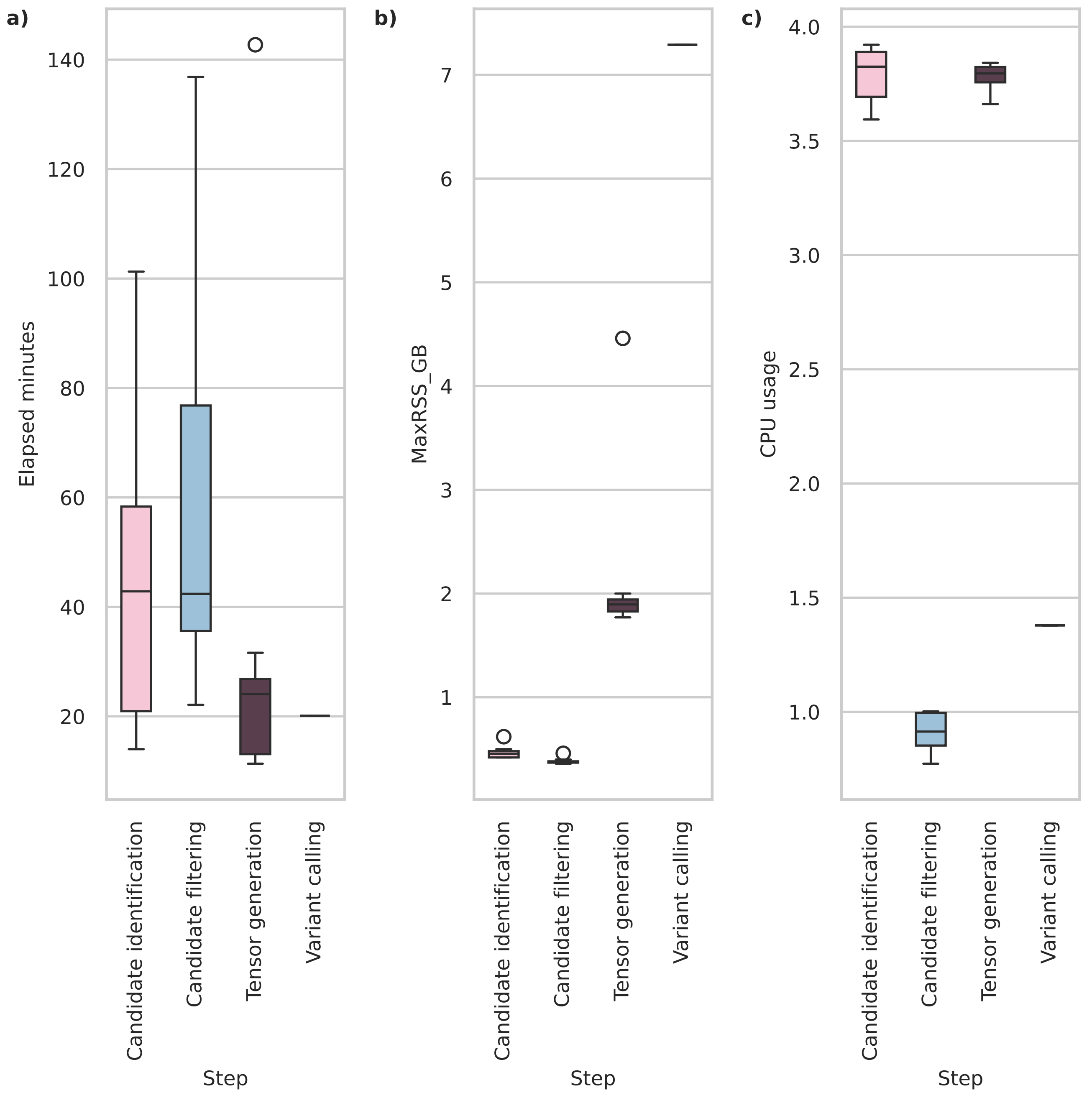

Figure S7: Runtime characteristics of pipeline phases across SEQC2-WES samples, showing (a) elapsed compute time per sample (minutes), (b) peak resident memory usage (MaxRSS, GB), and (c) effective CPU utilization (average cores used), aggregated at the pipeline phase level (Candidate identification, Candidate filtering, Tensor generation, Variant calling).

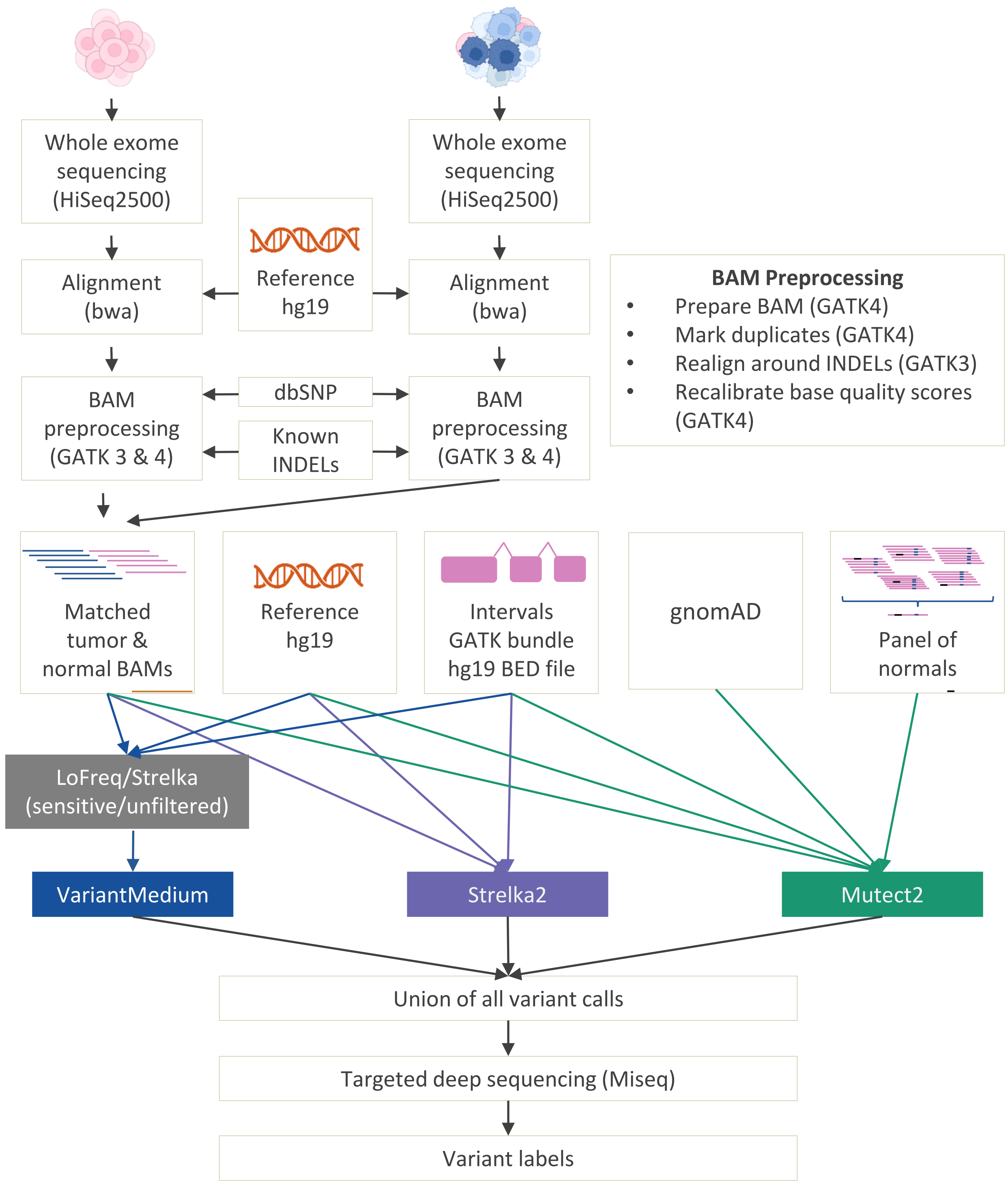

Figure S8: The calls produced by VariantMedium, Mutect2, and Strelka2 were experimentally confirmed through targeted deep sequencing. The cell line data were generated by whole-exome sequencing, and the reads were aligned to the hg19 reference genome using bwa. BAM preprocessing was performed with GATK3 and GATK4, incorporating dbSNP and known INDEL resources. Variants detected by any of the three callers—VariantMedium, Strelka2, or Mutect2—were subsequently deep sequenced on the MiSeq platform to obtain ground-truth labels.

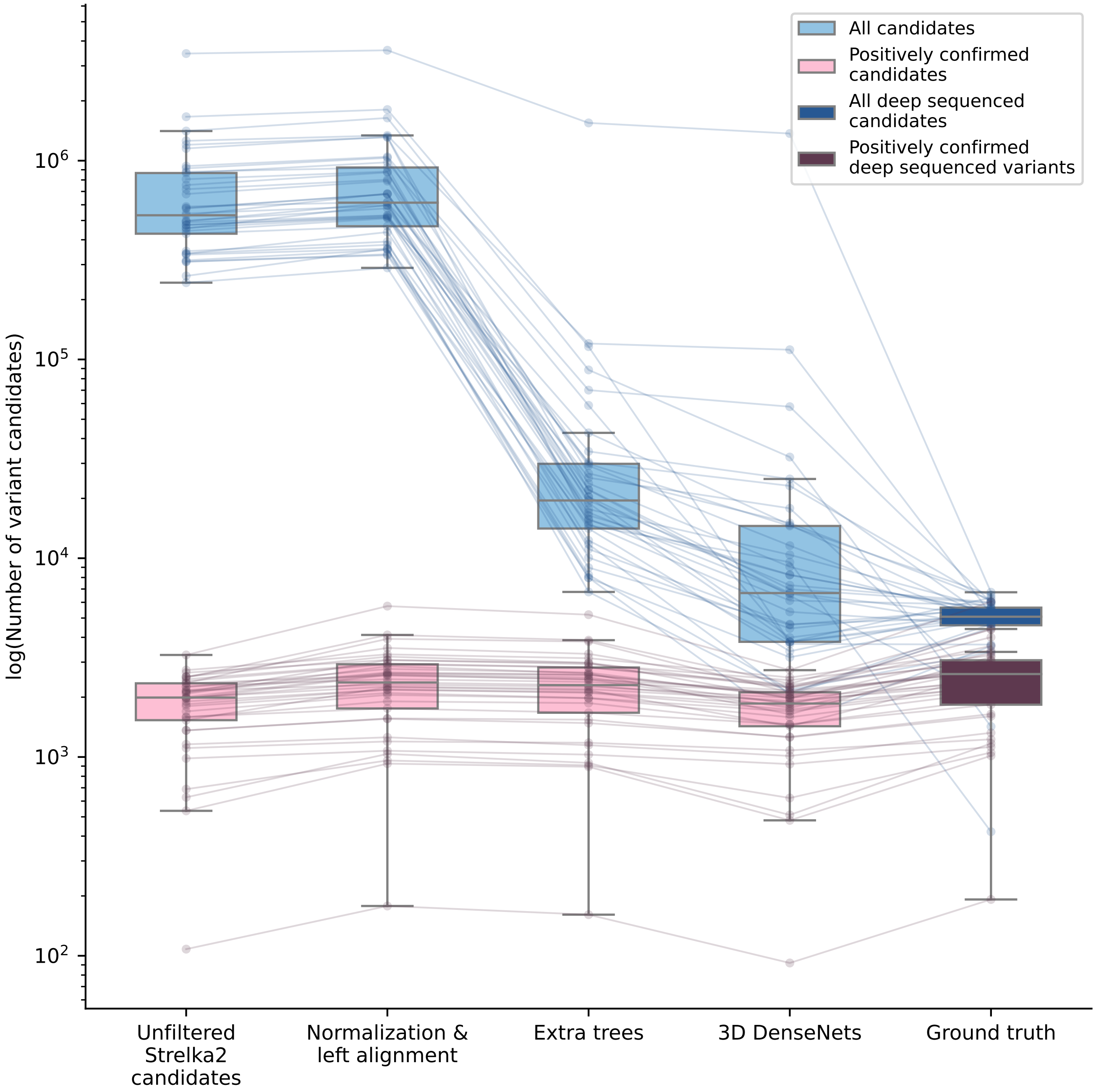

Figure S9: Number of candidate variants after each step of the pipeline, as well as the number of labels using data from 37 donors in the PCAWG-Pilot63 data set, shown for SNVs. VariantMedium workflow consists of multiple steps, starting with candidate identification using unfiltered Strelka2 candidates (PASS and non-PASS in the VCF file). Next step normalizes candidates, resolves biallelic substituions, and left aligns indels. Extra trees step does a prefiltering of candidates with very low support. 3D DenseNets makes the final decision on variant status. Ground truth is also shown here. Both the total number of candidates (in blue) and the candidates that are experimentally confirmed as somatic via targeted deep sequencing by PCAWG consortium is shown to demonstrate how much of the positive instances are lost at each step.

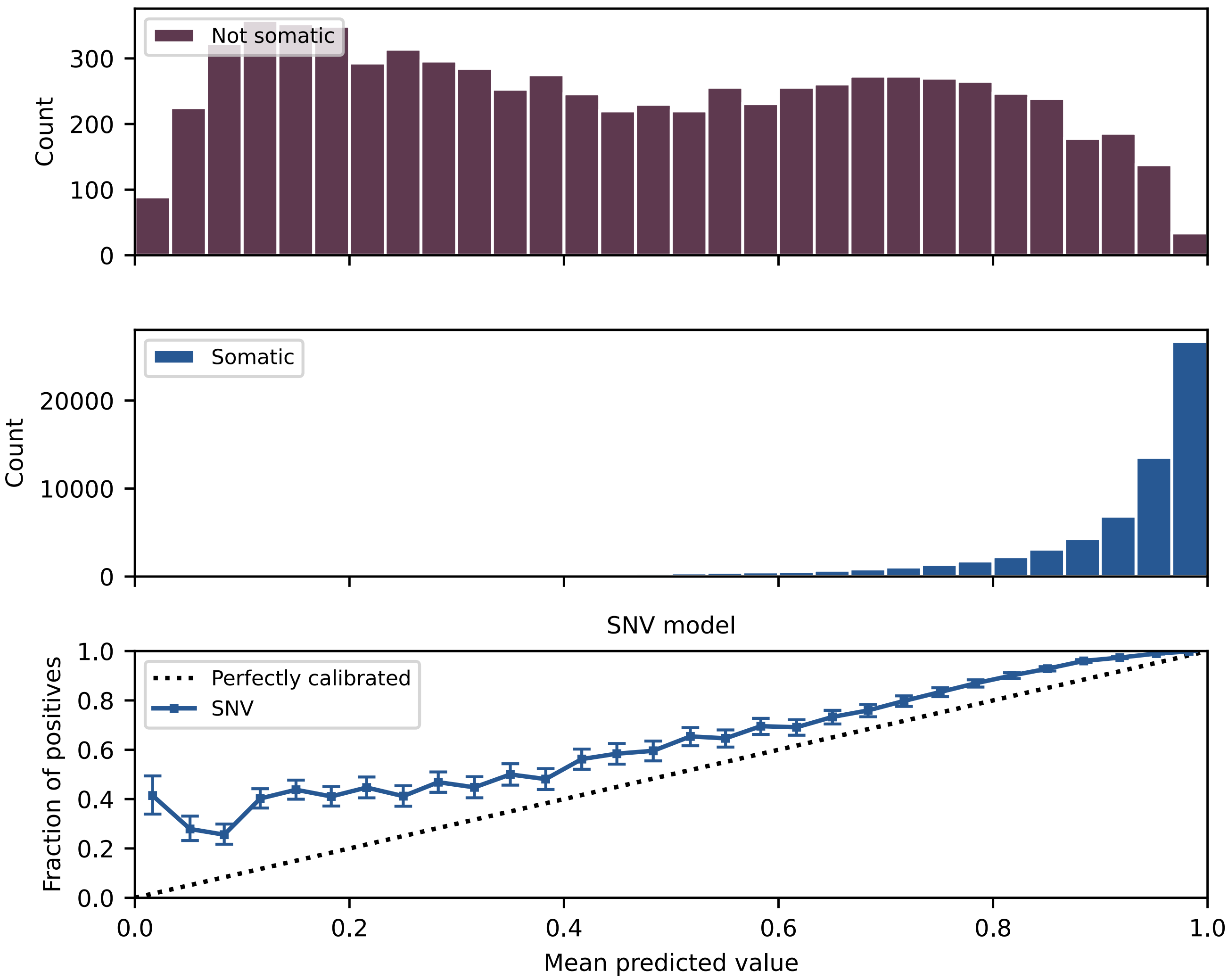

Figure S10: Calibration curve for the SNV model and the number of instances in each bin, shown for the targeted deep-sequenced candidate loci from 37 patients in the PCAWG-Pilot63 data set. The top row shows the histogram of negatively labeled instances, and the middle row shows the histogram of positively labeled instances. The bottom row presents the calibration in each bin based on the instances summarized in the top and middle rows.

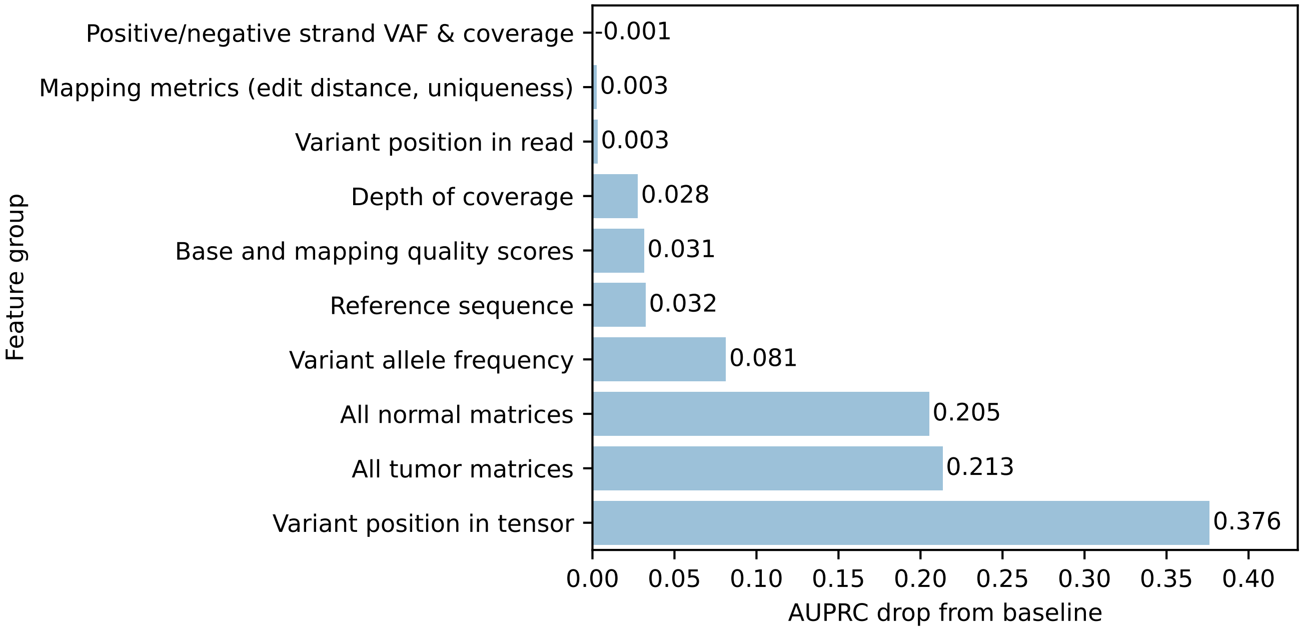

Figure S11: Feature-group importance derived from test-time ablation analysis. Bars show the decrease in average precision relative to the baseline model, where larger values indicate stronger dependence on the corresponding feature group. Ablation was performed by replacing the channels of each group with mean-based substitutes as described in the Methods, and importance values represent the resulting drop in predictive performance evaluated on the held-out test set.

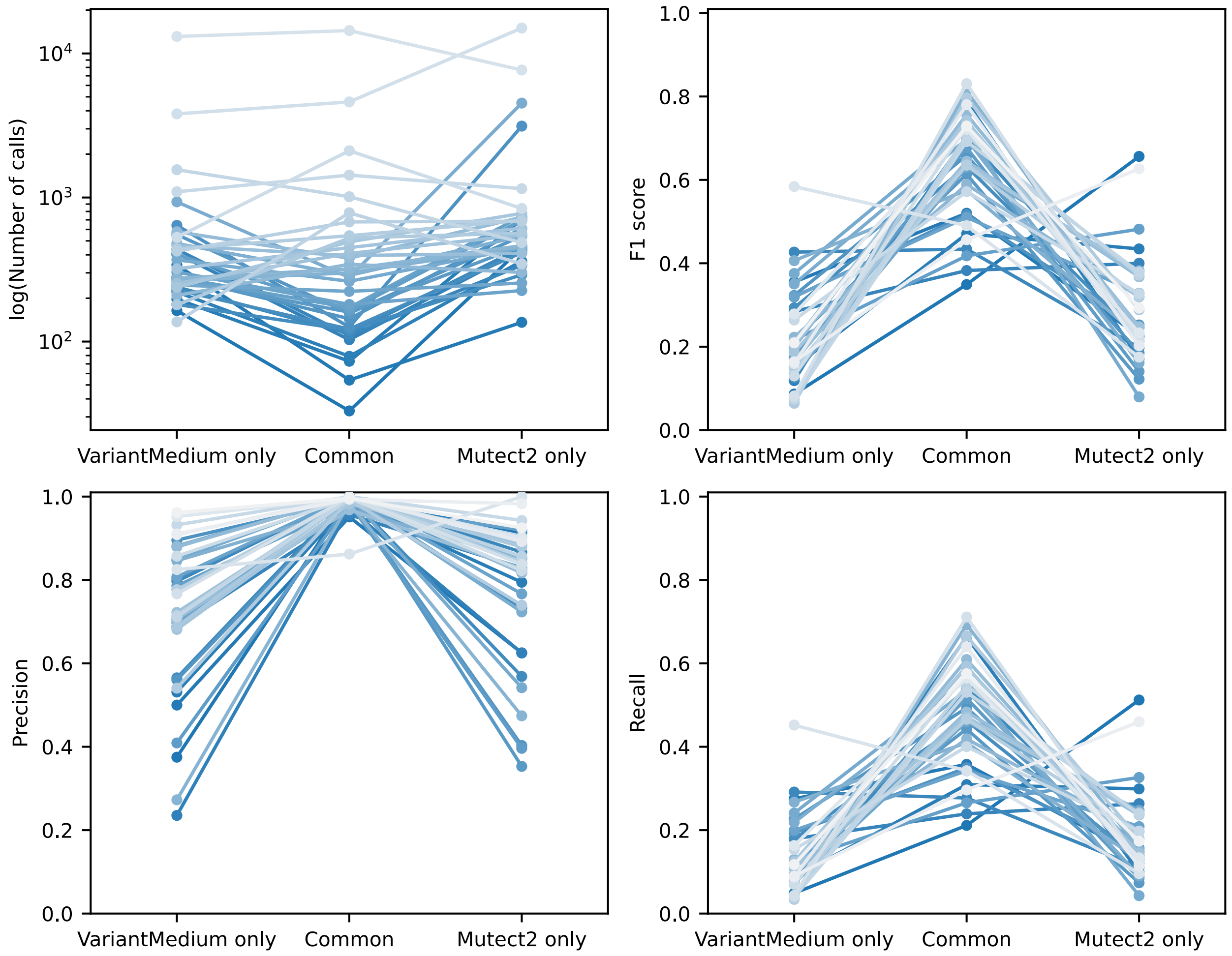

Figure S12: Comparison of VariantMedium and Mutect2 calls based on their common and unique candidate loci using data from 37 donors in the PCAWG-Pilot63 data set. For each method, we computed the number of candidate loci, as well as precision, recall, and F1-score values for the common and unique subsets. Unique VariantMedium calls refer to candidate loci called by VariantMedium but not by Mutect2, and unique Mutect2 calls refer to those called by Mutect2 but not by VariantMedium. The comparison is shown on a per-patient basis for SNVs; each line depicts a donor.

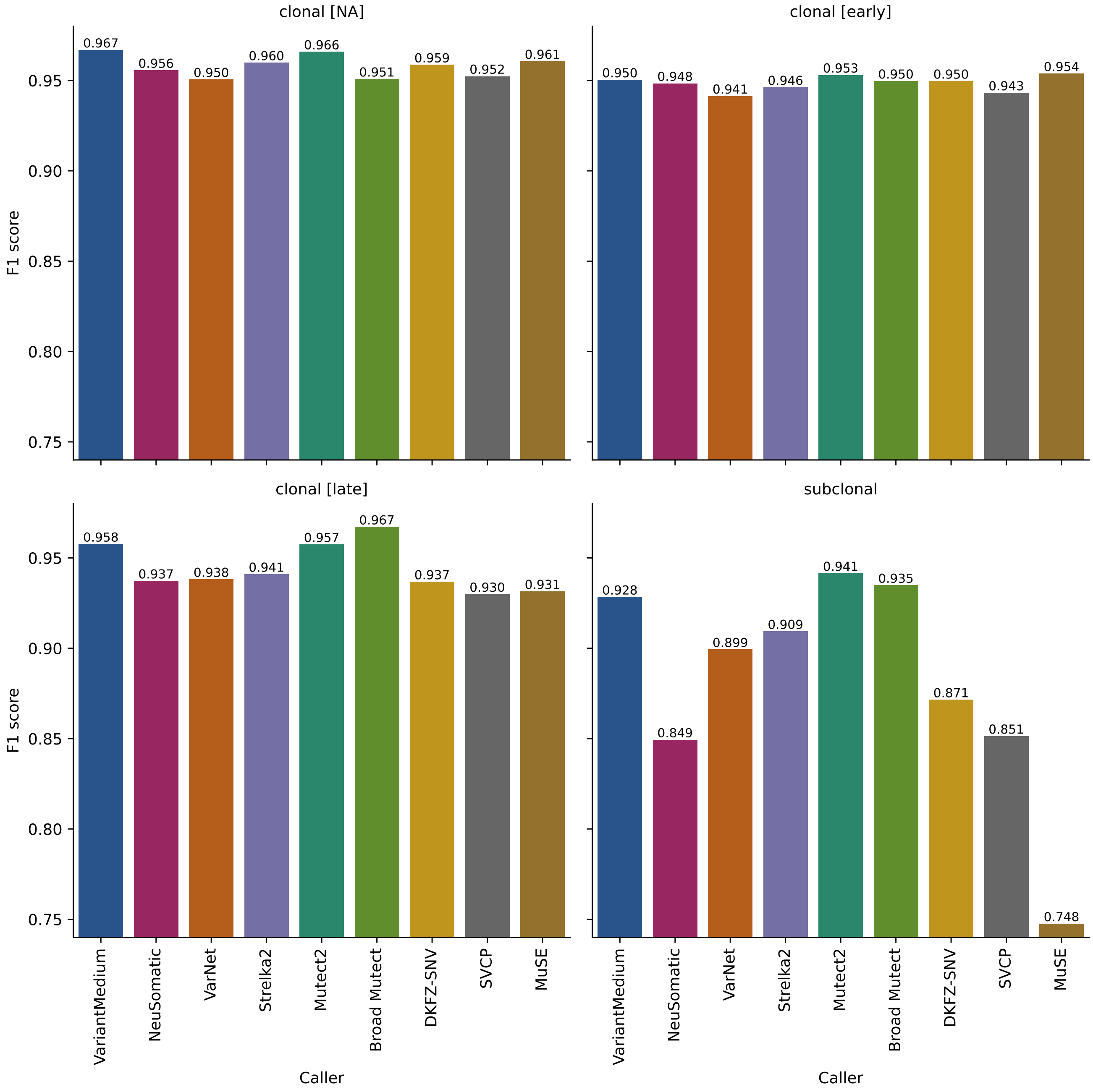

Figure S13: F1 scores of benchmarked variant callers across different clonality classes, using labeled data from 37 patients in the PCAWG-Pilot63 data set intersected with candidate loci for which clonality estimates are available according to the classification of the PCAWG Evolution and Heterogeneity Working Group and the PCAWG Consortium.

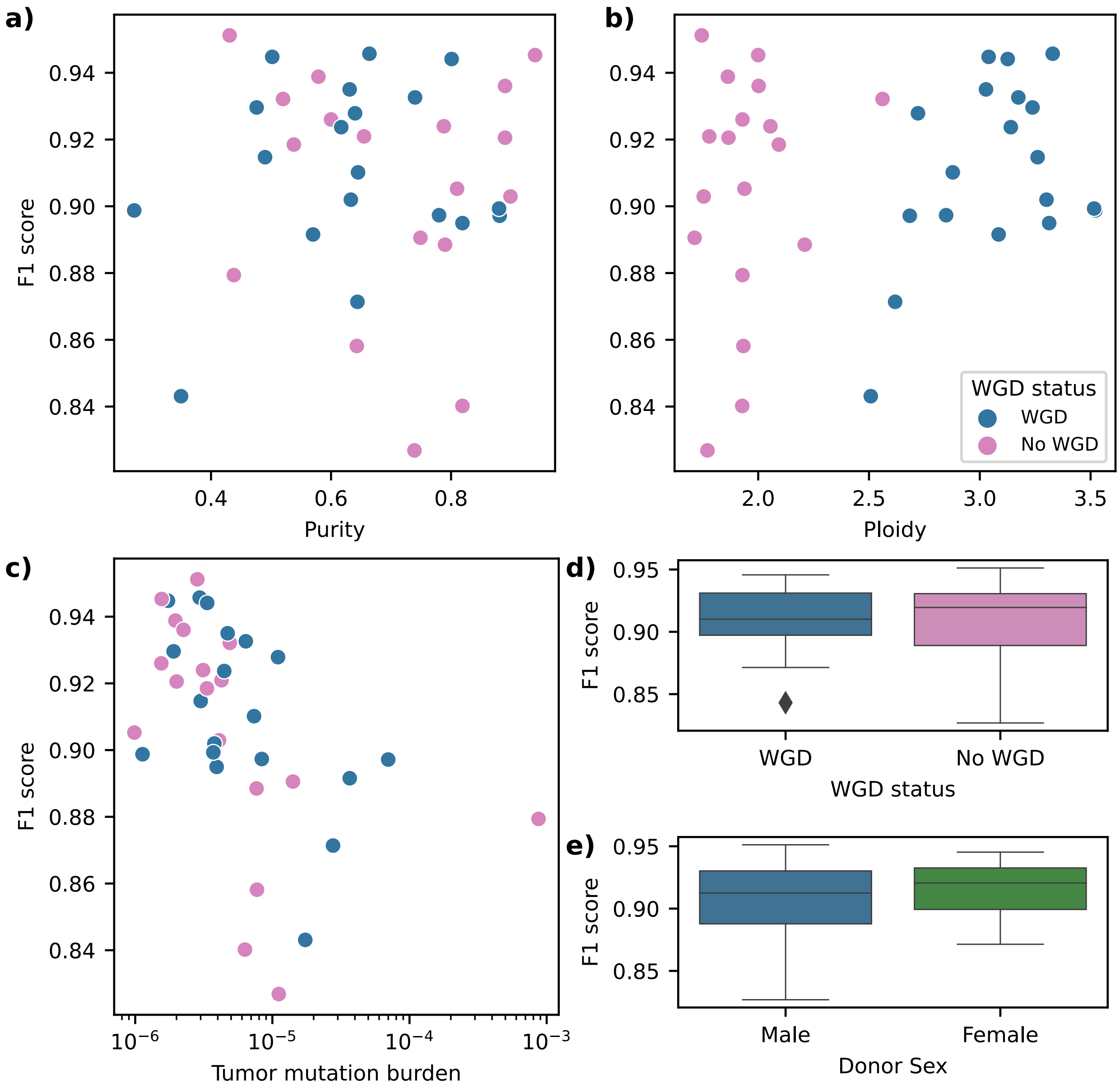

Figure S14: VariantMedium SNV calling performance in relation to sample purity, ploidy, whole-genome duplication (WGD), patient sex, and tumor mutational burden (TMB), based on data from 37 donors in the PCAWG-Pilot63 data set. Panels a–c show scatter plots of SNV F1 scores versus sample purity (a), sample ploidy (b), and TMB (c). Panels d–e show box plots of SNV F1 score distributions grouped by donor sex (d) and by WGD status (e).

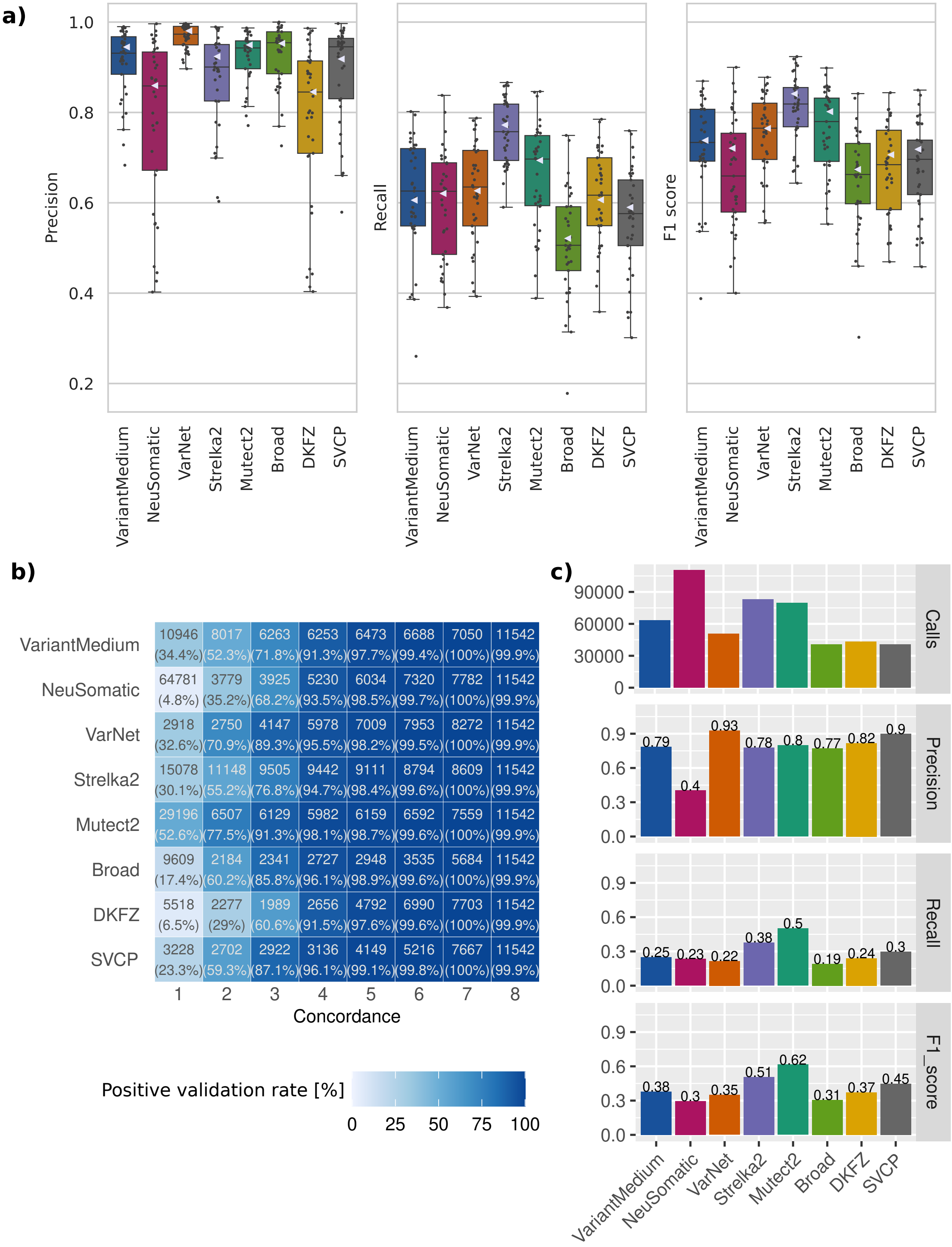

Figure S15: Somatic INDEL calling benchmark on PCAWG-Pilot63 data set. (a) Comparison of precision, recall, F1 score on labelled data. (b) Positive validation rates of tested tools based on the concordance bins. (c) Number of calls and precision, recall, F1 score calculated using concordance weighted performance comparison.

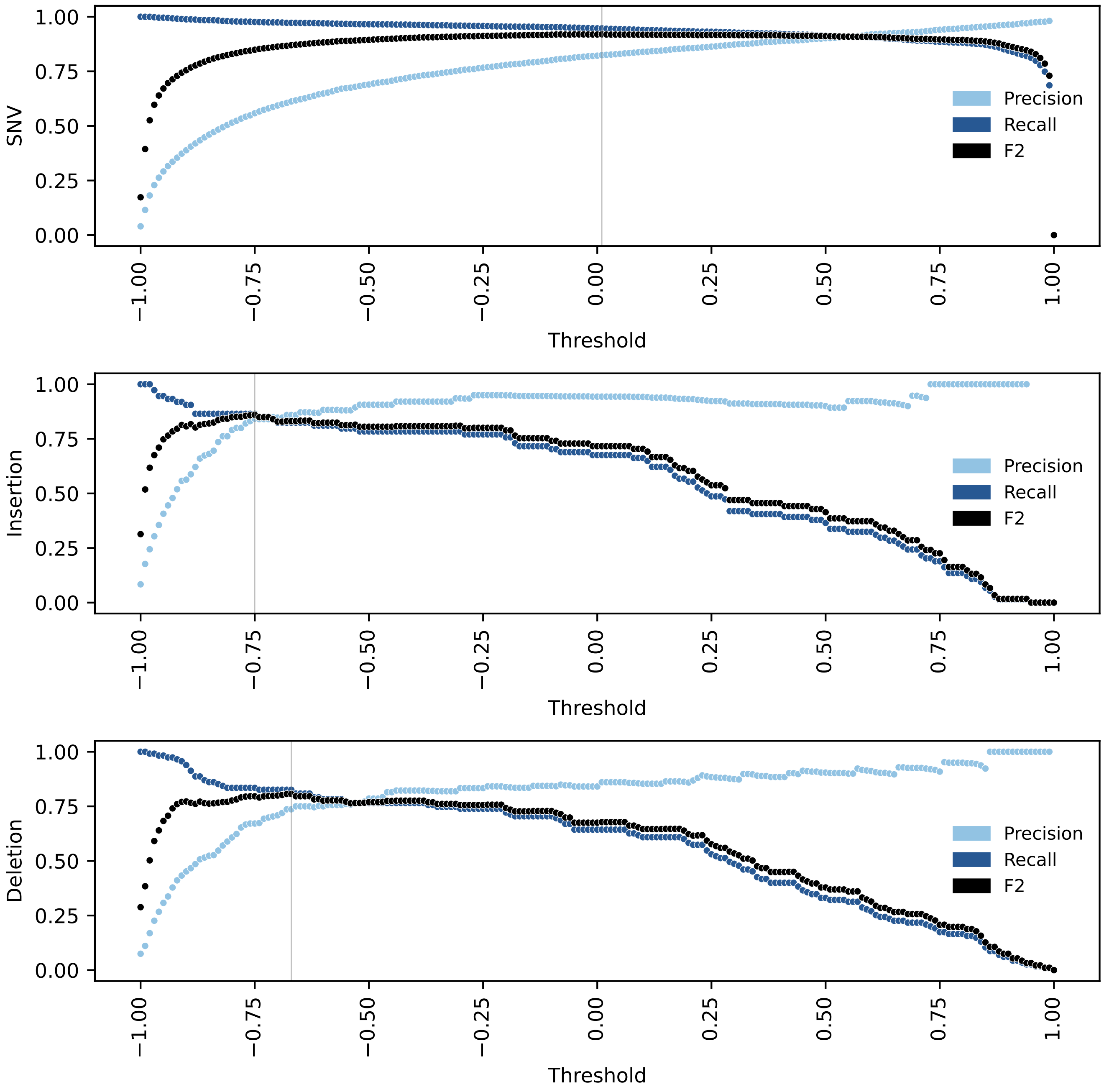

Figure S16: Impact of threshold choice on precision, recall, and F2 scores, and the thresholds used in VariantMedium to determine whether a candidate locus is classified as somatic or not. The panels show precision, recall, and F2 values computed on the validation set described in Supplementary Table 2, for SNVs, insertions, and deletions from top to bottom. The default thresholds correspond to the setting that maximizes the F2 score, assigning slightly greater weight to sensitivity. If needed, users may increase the threshold to obtain more precise results or decrease it to obtain more sensitive results. Users may also sort candidate loci by score and use the resulting ranking for prioritization.
